# Regulating Extra‐Framework Cations in Faujasite Zeolites for Capture of Trace Carbon Dioxide

**DOI:** 10.1002/chem.202201659

**Published:** 2022-07-13

**Authors:** Shanshan Liu, Yinlin Chen, Bin Yue, Chang Wang, Bin Qin, Yuchao Chai, Guangjun Wu, Jiangnan Li, Xue Han, Ivan da‐Silva, Pascal Manuel, Sarah J. Day, Stephen P. Thompson, Naijia Guan, Sihai Yang, Landong Li

**Affiliations:** ^1^ School of Materials Science and Engineering Nankai University Tianjin 300350 P. R. China; ^2^ Department of Chemistry The University of Manchester Manchester M13 9PL UK; ^3^ ISIS Facility STFC Rutherford Appleton Laboratory Chilton Oxfordshire OX11 0QX UK; ^4^ Diamond Light Source Harwell Science Campus Didcot Oxfordshire OX11 0DE UK; ^5^ Haihe Laboratory of Sustainable Chemical Transformations Tianjin 300192 P. R. China

**Keywords:** CO_2_ capture, extra-framework cations, faujasite, structure, structure-performance relationship

## Abstract

The development of cost‐effective sorbents for direct capture of trace CO_2_ (<1 %) from the atmosphere is an important and challenging task. Natural or commercial zeolites are promising sorbents, but their performance in adsorption of trace CO_2_ has been poorly explored to date. A systematic study on capture of trace CO_2_ by commercial faujasite zeolites reveals that the extra‐framework cations play a key role on their performance. Under dry conditions, Ba−X displays high dynamic uptake of 1.79 and 0.69 mmol g^−1^ at CO_2_ concentrations of 10000 and 1000 ppm, respectively, and shows excellent recyclability in the temperature‐swing adsorption processes. K−X exhibits perfect moisture resistance, and >95 % dry CO_2_ uptake can be preserved under relative humidity of 74 %. In situ solid‐state NMR spectroscopy, synchrotron X‐ray diffraction and neutron diffraction reveal two binding sites for CO_2_ in these zeolites, namely the basic framework oxygen atoms and the divalent alkaline earth metal ions. This study unlocks the potential of low‐cost natural zeolites for applications in direct air capture.

## Graphical abstract

**The explicit structure‐performance relationship** and underlying mechanism of trace CO_2_ capture by faujasite zeolites are elucidated. Two robust adsorbents are screened out, namely Ba−X with high dynamic CO_2_ uptake and K−X with unprecedented resistance to moisture.

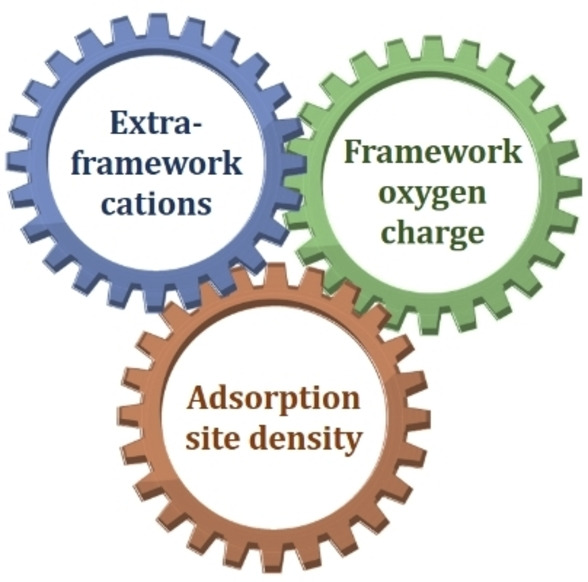

## Introduction

The increasing concentration of CO_2_ in the atmosphere has caused severe environmental problems,[^1^, ^2^] and efficient capture of CO_2_ has attracted much attention to meet the net‐zero targets. Currently, two main approaches for CO_2_ capture are: i) the large‐scale CO_2_ capture from pre‐combustion and post‐combustion gas streams; ii) removal of domestic CO_2_ by direct air capture (DAC).^[3]^ In addition, the removal of trace CO_2_ by‐product is essential in many industrial processes, such as the production of high‐purity CO and H_2_.^[4]^

Sorption‐based technologies are promising in promoting low‐cost and environmental‐friendly methods for CO_2_ capture. Among the various solid sorbents, natural or commercial‐available zeolites are low‐cost and highly stable and have been widely used for separation and catalysis in industry.[^5^, ^6^, ^7^] The adsorption of high‐concentration CO_2_ by zeolites has been studied thoroughly,[^8^, ^9^, ^10^, ^11^] and host‐guest interactions, molecular sieving effect, molecular ‘trapdoor’ and breathing effect are important features to optimise the uptake.[^12^, ^13^, ^14^] For the capture of trace CO_2_ (<1 %, from atmospheric environment, near emission source or enclosed space), strong basic sites in zeolites are required.^[15]^ Amino‐modified zeolites show strong affinity toward low concentrations of CO_2_ through the formation of carbonaceous species.^[16]^ However, heating is required for the regeneration of sorbents, resulting in high energy consumption and (partial) degradation of sorbents.

Criteria of an optimal sorbent for trace CO_2_ capture include: i) low‐cost and nil environmental or human toxicity; ii) high dynamic adsorption capacity greater than 1 mmol g^−1^; iii) good resistance to the presence of moisture; iv) moderate adsorption heats of 40–60 kJ mol^−1[17]^ and thus facile regeneration and high stability over long term. Recently, ion‐exchanged NaCa−A zeolites have shown high static adsorption of CO_2_ from gas mixtures with the concentration of CO_2_ being 400 (1.8 mmol g^−1^) to 5000 ppm (3.2 mmol g^−1^).^[19]^ However, the adsorption of CO_2_ on NaCa−A zeolites was irreversible, and the moisture resistance and regeneration process are yet to be investigated. Fu et al. reported that zinc containing chabazite (CHA) zeolites showed high dynamic uptake of 0.22 mmol g^−1^ for 400 ppm CO_2_, unlocking the potential of ion‐exchanged zeolites for DAC.^[18]^

Herein, we report a systematic study on capturing trace CO_2_ by alkali metals and alkaline earth metals (Li, Na, K, Cs, Mg, Ca, Ba) ion‐exchanged faujasite zeolites, aiming to establish the structure‐activity relationship to promote the development of practical sorbents for DAC. Under dry conditions, Ba−X exhibits high dynamic adsorption of CO_2_ of 1.79 and 0.69 mmol g^−1^ at concentration of 10000 and 1000 ppm, respectively, comparable with state‐of‐the‐art solid adsorbents. K−X exhibits perfect moisture resistance, and >95 % dry CO_2_ uptake can be preserved under relative humidity of 74 %. In situ synchrotron X‐ray powder diffraction, neutron powder diffraction, and solid‐state NMR spectroscopic studies reveal the key role of extra‐framework cations on trace CO_2_ capture. We propose general rules for the design of zeolite sorbents for their applications in DAC.

## Results and Discussion

M−X zeolites (M=Li, K, Cs, Mg, Ca, and Ba) were prepared via ion‐exchange from Na−X zeolite. The X‐ray diffraction (XRD) patterns and argon adsorption isotherms of as‐prepared M−X zeolites are shown in Figure S1 and S2, respectively, and their texture properties are summarised in Table S1. Li, K, Ca, Ba−X zeolites have achieved high exchange rate (>95 %) after two repeated ion‐exchange processes. Cs−X and Mg−X show low exchange rate (∼80 %) and reduction in the crystallinity, consistent with the previous reports.[^19^, ^20^, ^21^]

**Figure 1:**
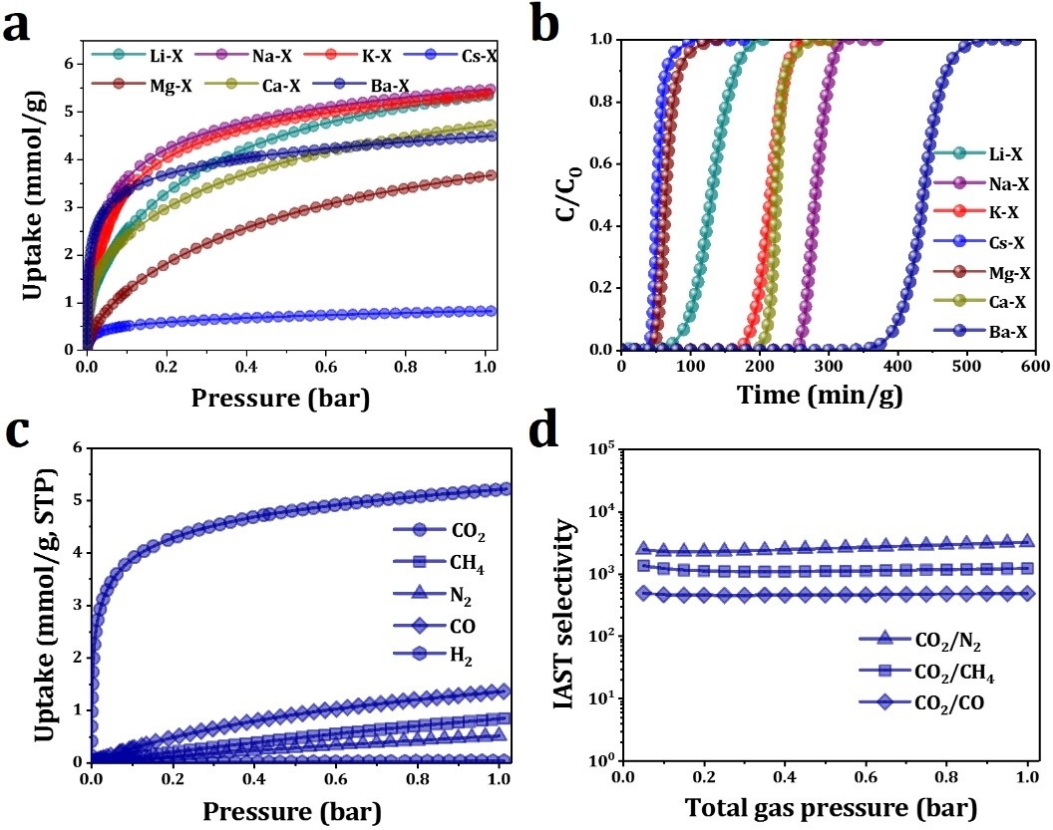
(a) CO_2_ adsorption isotherms on M−X zeolites at 298 K; (b) Dynamic gas breakthrough plots of 10,000 ppm CO_2_ in synthetic air over fixed‐bed packed with M−X zeolites. Conditions: 0.5 g adsorbent, 298 K, 1 bar and total flow rate of 10 mL/min. Only the CO_2_ breakthrough curves are shown for clarity; (c) CO_2_, CO, CH_4_, N_2_, O_2_ and H_2_ adsorption isotherms on Ba−X at 298 K; (d) Calculated IAST selectivity for CO_2_/CO, CO_2_/CH_4_ and CO_2_/N_2_ gas mixtures (1/99) on Ba−X.

CO_2_ adsorption isotherms of alkali metal‐exchanged and alkaline earth metal‐exchanged faujasite zeolites (M^I^‐X, M^II^‐X, respectively) were measured at 298 K and distinct profiles were observed (Figure 1a, Table S2).

At 1 bar, all M^I^‐X except Cs−X exhibit high adsorption capacities (>5.0 mmol g^−1^), followed by M^II^‐X (3–5 mmol g^−1^), and Cs−X (<1 mmol g^−1^). The volumetric adsorption capacity, which is an important parameter for practical applications in stationary capture and pressure swing adsorption (PSA) separation, is calculated. A high volumetric capacity of 233.7 cm^3^ cm^−3^ (4.46 mmol g^−1^) was achieved by Ba−X at 298 K and 1 bar (Table S3). At low pressure region, Ba−X shows the highest uptake of 108.8 cm^3^ cm^−3^ (2.09 mmol g^−1^) at 10 mbar, and of 37.0 cm^3^ cm^−3^ (0.71 mmol g^−1^) at 1 mbar among M−X zeolites (Figure S3, Table S2). These values are comparable to that of advanced adsorbents, such as SIFSIX‐3‐Ni, NbOFFIVE‐1‐Ni, SIFSIX‐18‐Ni‐β, NaCa−A and SIFSIX‐3‐Cu. (Table S4)[^2^, ^4^, ^17^, ^18^, ^19^, ^22^, ^23^, ^24^, ^25^] and thus indicates its great potential in trace CO_2_ capture.

The dynamic breakthrough experiments of M−X zeolites were conducted with low‐concentration of CO_2_, i. e., 1,000–10,000 ppm, in synthetic air at 298 K (Figure 1b and Table 1). In all experiments, the breakthrough of synthetic air (80 %N_2_–20 %O_2_) occurred in less than 1 minute, while CO_2_ was selectively retained. In this context, the dynamic selectivity calculated from the breakthrough curves are very high, typically >1,000. The dynamic uptake for 10,000 ppm CO_2_ follows the sequence of Ba−X (1.79 mmol g^−1^)>Na−X (1.15 mmol g^−1^)>Ca−X (0.92 mmol g^−1^)>K−X (0.88 mmol g^−1^)>Li−X (0.53 mmol g^−1^)>Mg−X (0.27 mmol g^−1^)>Cs−X (0.22 mmol g^−1^), which is consistent with the static isotherms. Similar sequences are obtained with CO_2_ concentrations of 1,000–5,000 ppm (Figure S4‐6, Table 1). Adsorption isotherms of the common components (CH_4_, CO, H_2_ and N_2_) in industrial carbon capture processes[^2^, ^6^] have been measured at 298 K on Ba−X (Figure 1c), confirming the notably higher uptake of CO_2_ in comparison with other gases. Ideal adsorbed solution theory (IAST) was employed to predict the adsorption selectivity, and the calculated IAST selectivity of Ba−X for mixtures (1/99) of CO_2_/N_2_, CO_2_/CH_4_ and CO_2_/CO are ∼2600, ∼1200, ∼500, respectively (Figure 1d, Figure S7‐12). The high selectivities illustrate the potential of M−X zeolites, especially Ba−X, in capturing trace CO_2_ from a variety of mixtures.

**Table 1:** Breakthrough data of M−X zeolites in CO_2_ capture.

Zeolites	Dynamic uptake [mmol g^−1^]				Humidity
	10,000 ppm	5,000 ppm	3,000 ppm	1,000 ppm	
Li−X	0.53	0.3	0.23	0.08	dry
	0.39	0.19	0.17	0.07	74 % RH
Na−X	1.15	1.14	0.93	0.41	dry
	0.70	0.65	0.55	0.29	74 % RH
K−X	0.88	0.71	0.50	0.33	dry
	0.88	0.70	0.49	0.29	74 % RH
Cs−X	0.22	0.17	0.14	0.08	dry
	0.21	0.15	0.13	0.06	74 % RH
Mg−X	0.27	0.16	0.11	0.06	dry
	0.26	0.10	0.10	0.04	74 % RH
Ca−X	0.92	0.75	0.61	0.44	dry
	0.87	0.60	0.54	0.25	74 % RH
Ba−X	1.79	1.42	1.25	0.69	dry
	1.56	1.20	0.95	0.45	74 % RH

**Figure 2:**
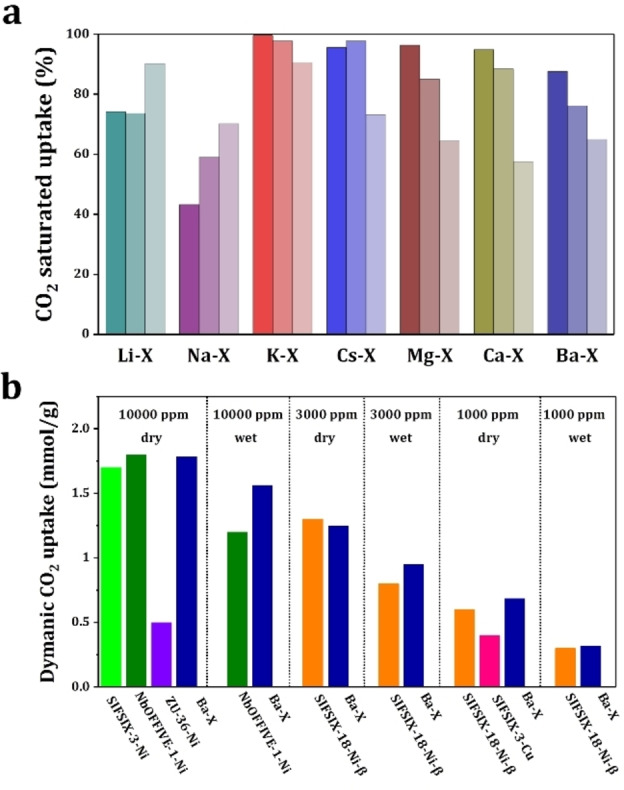
(a) Bar diagram showing the relative decrease of CO_2_ dynamic uptake (1,000, 3,000 or 10,000 ppm CO_2_ from light to dark) in M−X zeolites in the presence of moisture (74 % RH); (b) Comparison of dynamic CO_2_ uptake between Ba−X and the state‐of‐the‐art solid adsorbents from literature under different conditions.

The impact of operating parameters on trace CO_2_ capture in Ba−X were investigated. The dynamic uptake of CO_2_ in Ba−X decreases with increasing temperature from 298 to 323 K (Figure S13) or diluting the CO_2_ concentration to 400 ppm (close to the average concentration in the atmosphere) (Figure S14). Interestingly, Ba−X still shows high adsorption capacity at 333 K (55.8 cm^3^ cm^−3^ for 10,000 ppm CO_2_) or with 400 ppm CO_2_ (16.7 cm^3^ cm^−3^ at 298 K). It is widely acknowledged that the present of moisture could have detrimental effects for CO_2_ capture due to the competing adsorption of water.[^9^, ^10^, ^26^, ^27^, ^28^] Herein, the performance of M−X zeolites for trace CO_2_ capture in the presence of moisture (74 % relative humidity, RH) was studied by breakthrough experiments of 1,000–10,000 ppm CO_2_ in synthetic air (Figure S15–18 and Table 1). The impact of moisture is summarized in Figure 2a and appears to be dependent on the type of extra‐framework cations of these zeolites. Alkaline earth metals, with greater chemical hardness, show high affinity towards water, and thus M^I^‐X shows better water resistance than M^II^‐X. K−X is almost unaffected by the presence of moisture and 95 % of the dry CO_2_ uptake is preserved. By contrast, 65 % of the dry CO_2_ uptake was retained with Ba−X, which however still shows the best breakthrough performance among all M−X zeolites under ‘wet’ conditions (Table 1). The dynamic uptakes of 1,000–10,000 ppm CO_2_ on Ba−X under both dry and wet conditions are lower than that of the best‐performing MOFs, such as NbOFFIVE‐1‐Ni@PA, Zn‐(ZnOH)_4_(bibta)_3_, but comparable with those of non‐amino functionalised adsorbents (Figure 2b, Table 1, Table S4).[^2^, ^4^, ^17^, ^22^, ^25^] To date, it is acknowledged that Na−X form the current benchmark for CO_2_ sorbents.[^29^, ^30^] The working capacity of Ba−X is increased to 1.7 times of Na−X and the moisture resistance is greatly improved. Ba−X is a transition metals‐free sorbent with a highly competitive price of <$3000 per tonne, compared with Zn‐CHA and MOFs.^[18]^

**Figure 3:**
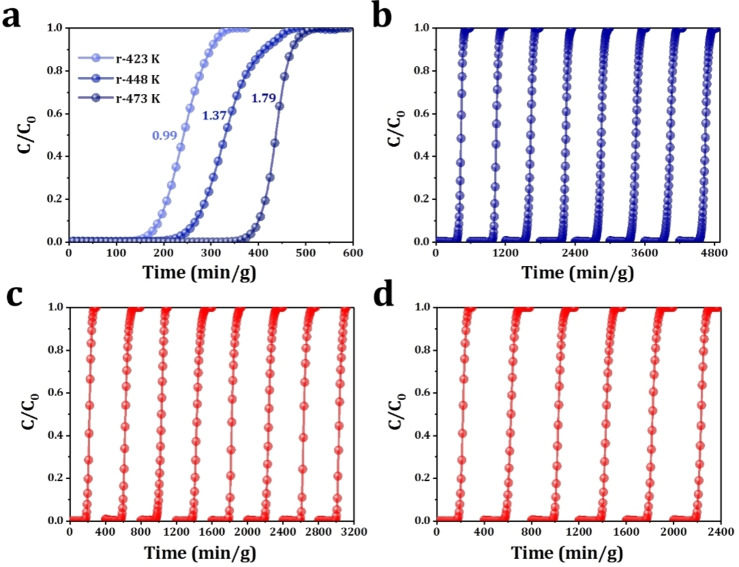
(a) Dynamic gas breakthrough tests of 10,000 ppm CO_2_ in synthetic air with on Ba−X zeolites regenerated by heating to 423 (r‐423k), 453 (r‐453k) and 473 K (r‐473k), respectively. Conditions: 0.5 g adsorbent, 298 K, 1 bar and total flow rate of 20 mL/min; (b) Recyclability of Ba−X in the breakthrough tests of 10000 ppm CO_2_ in synthetic air. Adsorbent regenerated by heating to 473 K at a rate of 10 K/min; Recyclability of K−X in the breakthrough tests of 10000 ppm CO_2_ in synthetic air under dry (c) and wet (d) conditions. 74 % RH for wet conditions, adsorbent regenerated by heating to 473 K at a rate of 10 K/min.

The recyclability of these zeolites was also investigated. From the temperature‐programmed desorption (TPD) profiles of CO_2_ (Figure S20), multiple CO_2_ desorption peaks were observed in the temperature range of 323–423 K, indicating the complete removal of adsorbed CO_2_ by thermal treatment at >423 K. For Ba−X zeolite, thermal regeneration was performed at 423 K, 448 K and 473 K and the CO_2_ breakthrough curves (10,000 ppm in synthetic air) were examined. As shown in Figure 3a, full regeneration of Ba−X can be achieved by heating at 473 K for 20 minutes and the dynamic CO_2_ uptake was fully retained along eight cycles of breakthrough experiments (Figure 3b). The regeneration performance of Ba−X is superior to NaCa‐A^[19]^ due to the absence of strongly irreversibly‐adsorbed species. The above results clarify that Ba−X is an ideal trace CO_2_ adsorbent realizing the optimization of cost, adsorption capacity and regeneration. Excellent recyclability under both dry (Figure 3c) and wet conditions (Figure 3d) was also observed on K−X with perfect moisture resistance, which demonstrate that Ba−X and K−X are efficient sorbents for trace CO_2_ capture applicable in temperature‐swing adsorption processes.

**Figure 4:**
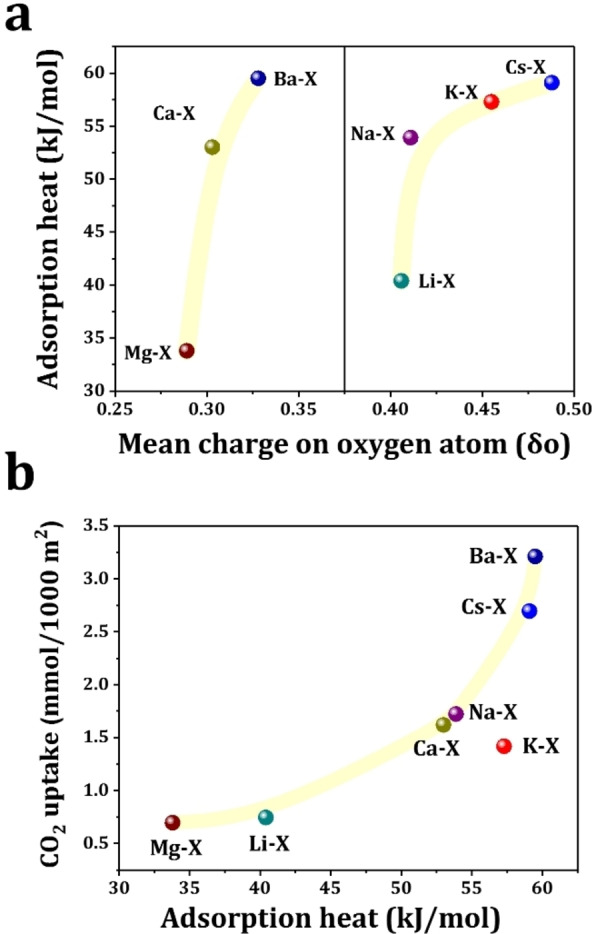
(a) Correlation between the calculated mean charge on framework oxygen atom and the measured Q_st_ of CO_2_ adsorption on M−X zeolites; (b) Correlation between the measured Q_st_ of CO_2_ adsorption and dynamic CO_2_ (10,000 ppm) uptake in M−X zeolites per 1000 m^2^ microporous surface area.

Isosteric heats of adsorption (*Q*_st_) for CO_2_ uptakes on M−X zeolites were measured by differential scanning calorimetry (DSC) (Figure S21–27). The *Q*_st_ value of Cs−X (59.1 kJ mol^−1^)>K−X (57.3 kJ mol^−1^)>Na−X (53.9 kJ mol^−1^)>Li−X (40.4 kJ mol^−1^) is obtained with M^I^‐X, Ba−X (59.5 kJ mol^−1^)>Ca−X (53.0 kJ mol^−1^)>Mg−X (33.8 kJ mol^−1^) with M^II^‐X. The adsorption heats determined from the Henry constants using van′t Hoff equation show the identical trend as that measured by DSC (Table S6, Figure S28–33).^[31]^ The introduction of different cations may cause changes in unit cell weight, framework crystallinity and pore accessibility, thus affecting the diffusion and adsorption of CO_2_. To eliminate these effects, the CO_2_ uptake of M−X is normalized by the microporous surface area of the samples as CO_2_ adsorption occurs dominantly on the microporous structure (Table S5). The correlation between the dynamic CO_2_ uptake per microporous surface area (1000 m^2^) and the *Q*_st_ of the adsorption could indicates that the balance between the adsorption strength and microporous surface area plays the key role in the capture of trace CO_2_ with M−X zeolites. (Figure 4b). CO_2_ can interact with Lewis‐basic oxygen sites on the zeolite as an acidic gas, and it can also bond to metal cations via its oxygen centre. The basicity of the framework can influence the strength of both physisorption and chemisorption, and the latter is also affected by the hardness of the metal. The average basic strength of framework oxygen sites can be calculated by their mean charge δ_O_ evaluated from Sanderson's electronegativity equalization principle (Table 2).[^32^, ^33^, ^34^]

**Table 2:** Characteristics of framework oxygen charge on M−X zeolites.

Zeolites	unit cell composition	Intermediate electronegativity (S_int_)	Mean charge on oxygen atom (δ_O_)
Li−X	Li_86_(Al_86_Si_106_O_384_)	3.288	0.406
Na−X	Na_86_(Al_86_Si_106_O_384_)	3.260	0.411
K−X	K_86_(Al_86_Si_106_O_384_)	3.051	0.455
Cs−X	Cs_86_(Al_86_Si_106_O_384_)	2.894	0.488
Mg−X	Mg_43_(Al_86_Si_106_O_384_)	3.836	0.289
Ca−X	Ca_43_(Al_86_Si_106_O_384_)	3.771	0.303
Ba−X	Ba_43_(Al_86_Si_106_O_384_)	3.655	0.328

As shown by the δ_O_ data, the average basic strength of M−X follows the trend: Cs−X>K−X>Na−X>Li−X, and Ba−X>Ca−X>Mg−X, which is consistent with the trend of Q_st_ (Figure 4a). The extra‐framework cations can tune the alkalinity of the framework oxygen atoms to adjust CO_2_ adsorption strength. The basic strength declines significantly after exchange with divalent alkaline earth cations, as observed in a previous study.^[35]^ However, the adsorption capacity of Ba−X and Ca−X is very high, especially for Ba−X, indicating the presence of other factors that influence the CO_2_ adsorption on CO_2_ capture. (Figure S34–49, additional discussion shown in the Supporting Information).

Through comprehensive analyses of these results on CO_2_ adsorption with M−X zeolites, the following rules can be reached. i) The surface‐specific dynamic CO_2_ uptake is closely related to the adsorption strength (*Q*_st_), and the simultaneously high adsorption strength and microporous surface area are key to achieve high CO_2_ adsorption. ii) *Q*_st_ is related to the basicity of the framework oxygen atoms, and extra‐framework cations can efficiently regulate the basicity of framework oxygen atoms. iii) Although divalent alkaline earth metal ions in M−X zeolites reduce the average alkalinity of framework oxygen atoms (in comparison with monovalent alkali metal ions), they promote dynamic CO_2_ uptake likely by creating new adsorption sites. iv) The employment of large monovalent alkali metal ions, such as K^+^, may provide a solution to mitigate the adverse effects of moisture.

Na−X and Ba−X are selected as models to reveal the host‐guest interactions in M^I^‐X and M^II^‐X upon adsorption of CO_2_ and to probe the presence of additional sites in M^II^‐X. Both structures were determined from refinement of neutron powder diffraction (NPD) or synchrotron X‐ray powder diffraction data with excellent agreement factors (Figure S50–53, Table S10). It is worth noting that the determination of binding sites for CO_2_ at low loading (<1 %) is subject to large uncertainties and thus the host‐guest interactions have been studied at high loadings of CO_2_.

**Figure 5:**
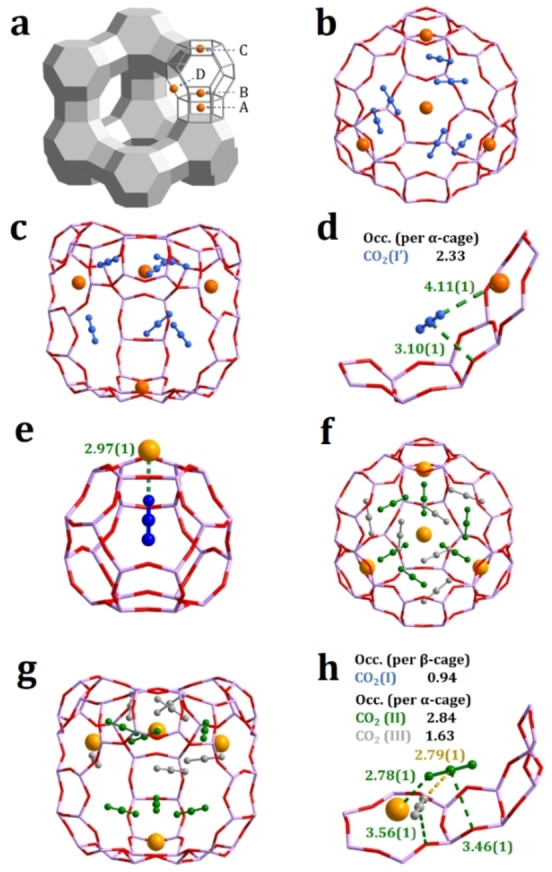
Cation distribution in faujasite zeolite (a) and CO_2_ adsorption sites in Na−X (b‐d) and Ba−X (e–h) from NPD and synchrotron XRD Rietveld refinement. For clarity, guest molecules at sites I, II and III are coloured and highlighted with the occupancies in blue, green, and gray, respectively. (Si/Al: lavender; O: red; Na/Ba: orange)

The exchanged cations, which balanced the negative charge, usually occupy some or all specific sites in the structure^[36]^ (Figure 5a). In Na−X, Na^+^ occupied all four sites and left merely the α‐cage unoccupied. Only one CO_2_ site was found in this cage, interacting to the framework oxygen with an electrostatic interaction (C_CO2_⋅⋅⋅O_framework_=3.10(1) Å) and to the site D cation (O_CO2_⋅⋅⋅Na=4.11(1) Å), affording an uptake of 2.33 CO_2_ per α‐cage. In Ba−X, due to the high valence of Ba^2+^, the cation only occupied site A and D and thus effectuate the β‐cage for adsorption. The CO_2_ in β‐cage at site I formed a strong interaction to the site D cation (O_CO2_⋅⋅⋅Ba=2.97(1) Å), with a nearly full occupancy of 0.94 per β‐cage, which is regarded as the preferred site of adsorption. Besides, two types of CO_2_ are found in the α‐cage, site II and III: site II interacts with the site D cation (O_CO2_⋅⋅⋅Ba=2.78(1) Å) and with the framework oxygen (C_CO2_⋅⋅⋅O_framework_=3.46(1) Å); site III exhibits a dipole interaction with site II (C_CO2_⋅⋅⋅O_CO2_=2.79(1) Å).

**Figure 6:**
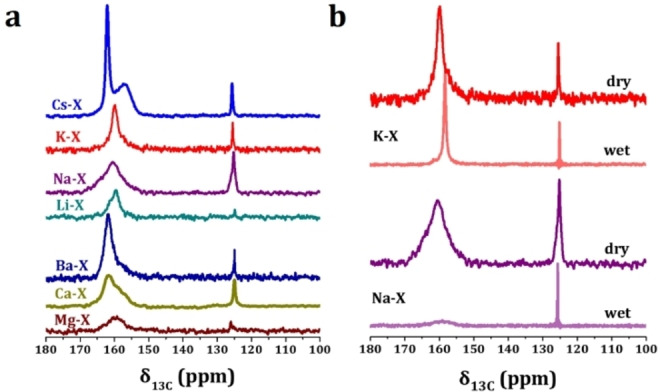
(a) ^13^C CP/MAS NMR spectra of ^13^CO_2_ adsorption on M−X zeolites; (b) ^13^C CP/MAS NMR spectra of ^13^CO_2_ and H_2_O co‐adsorption on Na−X and K−X zeolites.

The underlying mechanism of CO_2_ adsorption on M−X zeolites was also investigated by in situ solid‐state nuclear magnetic resonance (NMR) spectroscopy (Figure 6a), which reveals the presence of both physisorbed (in the region of 120–130 ppm) and chemisorbed species (in the region of 150–170 ppm) upon feeding ^13^CO_2_ to M−X zeolites.[^37^, ^38^, ^39^] Stronger chemisorption, as reflected by the shift of ^13^C signals toward higher field, can be observed with higher alkalinity of framework oxygen atoms. With the co‐feeding of ^13^CO_2_ and H_2_O to Na−X, the signal due to chemisorbed ^13^CO_2_ species almost disappeared and the physisorbed ^13^CO_2_ species is preserved, consistent with the negative impact of moisture on dynamic CO_2_ uptake (Figure 2a, Table 1). In contrast, the presence of moisture weakened slightly the chemisorption of ^13^CO_2_ on K−X (^13^C signal shifted toward lower field by 2 ppm) and most of chemisorbed species are preserved, confirming the moisture resistance of K−X in the breakthrough studies. Cu^2+^‐exchanged SSZ‐13 has been reported as an ideal adsorbent for CO_2_/N_2_ separation in the presence of moisture.^[40]^ Fe‐containing mordenite with the precisely narrowed channels of ∼3.3 Å exhibits good moisture resistance in CO_2_ adsorption.^[12]^ Cu‐SSZ‐13, Fe‐ mordenite and K−X all have large metal ions or species (Cu^2+^, isolated tetrahedral Fe species or K^+^, in comparison with the pore size of zeolites) that can act as the primary binding sites for H_2_O.^[40]^ Under such circumstances, the competition on the basic sites (framework oxygen atoms) with CO_2_ can be suppressed and the negative impacts from co‐existing moisture minimised.

## Conclusion

Sorption‐based CO_2_ capture is an important technology to remove low‐concentration CO_2_ from atmospheric environment, near emission source and enclosed space. Natural or commercial‐available zeolites with intrinsic stability and low‐cost characteristic are promising candidates for DAC. In this work, we have conducted a comprehensive study on trace CO_2_ capture by faujasite zeolites to reveal the role of extra‐framework cations. Two efficient adsorbents have been identified, namely Ba−X with high dynamic CO_2_ uptake and K−X with unprecedented resistance to moisture. The overall performance of Ba−X can satisfy the basic requirements for practical trace CO_2_ capture coupled with temperature‐swing adsorption processes. The surface‐specific dynamic CO_2_ uptake closely related to the adsorption strength, which is rationally related to the basicity of the framework oxygen atoms. The framework oxygen atoms of alkali metal and alkaline earth metal ion exchanged faujasite zeolites are determined as the primary sites for CO_2_ adsorption via simple acid‐base interaction, and significant contribution from divalent alkaline earth metal ions such as Ba^2+^ has also been confirmed. Although faujasite zeolite are known as promising CO_2_ adsorbent for decades, a revisit to such system herein unlocks its potential in trace CO_2_ capture and the explicit structure‐performance relationship is most helpful for the development of robust adsorbents.

## Experimental Section

**Preparation of samples**: Commercial Na−X and Na−Y zeolites were purchased from Sinopec Co. and employed as the parent zeolites. M−X and M−Y zeolites were prepared from Na−X and Na−Y, respectively, via ion‐exchange in the aqueous phase. In a typical experiment, 3.0 g of commercial Na−X (Si/Al=1.2) or Na−Y (Si/Al=5.3) zeolites was ion‐exchanged with 100 mL of 1.0 M nitrate {LiNO_3_, KNO_3_, Mg(NO_3_)_2_ or Ca(NO_3_)_2_}solution under stirring at 353 K for three times (the concentration of Ba(NO_3_)_2_ was 0.2 M due to its low solubility in water). After each ion‐exchange process, the slurry was filtered and washed with deionized water. The final solid product was dried at 353 K overnight and calcined in flowing dry air at 573 K for 6 h to obtain final products. Since the diameter of Cs^+^ ion is relatively large, to increase the exchange level, Cs−Y and Cs−X were prepared from K−Y and K−X, respectively. The partial framework collapse and instability of Mg−X are originated from the formation of MgO during the calcination process, which also limits the exchange level to 86 % after repeated ion‐exchange processes. The large volume of Cs^+^ (ionic diameter ∼3.6 Å) leads to framework instability and crystallinity decline of Cs−X, and the exchange level is limited to 80 % due to the inaccessibility of bulky Cs^+^ to certain extra‐framework cation positions.

**Characterization of zeolites**: The powder X‐ray diffraction (XRD) patterns of the samples were recorded on a Bruker D8 diffractometer using Cu−Kα radiation (λ= 0.1541 nm) at a scanning rate of 6°/min in the region of 2θ=5‐50°. The surface areas of zeolite samples were calculated from argon adsorption and desorption isotherms at 87 K collected on a Quantachrome autosorb iQ gas adsorption instrument. The total surface area was calculated via the Brunner Emmett Teller (BET) equation as built in the software. The chemical compositions of zeolite samples were analyzed on an IRIS Advantage inductively coupled plasma atomic emission spectrometer (ICP‐AES).

**Gas Adsorption Measurements**: The CO_2_, CO, CH_4_, N_2_, O_2_ and H_2_ sorption isotherms were measured on a Beishide BSD‐PMC gas adsorption instrument. Prior to the gas adsorption measurements, the samples were activated at 573 K under dynamic vacuum for 6 h to generate fully desolvated zeolite samples. A circulator bath was used to maintain the required temperature.

**Column breakthrough studies**: Column breakthrough experiments were conducted on a customer‐built fixed‐bed packed with ∼0.5 g samples. Before the breakthrough experiment, the samples were treated by heating to 523 K in a flow of argon at 30 mL/min for 30 min to remove trace adsorbates in zeolites such as water. After cooling to 298 K, a CO_2_/N_2_ mixture (10000 ppm, 5000 ppm, 3000 ppm and 1000 ppm) was fed to the adsorber. The outlet composition was on‐line monitored by a mass spectrometer (MS). Experiments in the presence of moisture were performed by passing the gas stream through a water vapor saturator at 298 K to obtain the relative humidity (RH) of 74 %.

**IAST analysis of the selectivity data of hydrocarbon adsorption**: Ideal adsorbed solution theory (IAST) was used to predict the selectivity factor for binary mixtures using pure component isotherm data [Equation (1)]. The selectivity factor, S , is defined according to the equation below where $x_{i}$ is the amount of each component adsorbed as determined from isotherms and $y_{i}$ is the mole fraction of each component in the gas phase at equilibrium.

(1) $$S=\frac{x_{1}y_{2}}{x_{2}y_{1}}\;$$

**Isosteric heat of adsorption**: Isosteric adsorption heat for CO_2_ on the zeolite samples were measured using a Q600 SDT simultaneous thermal analyzer (TA Instruments). In a typical experiment, ∼10 mg of the sample was pre‐treated at 573 K under a flow of helium at the rate of 100 mL/min. After cooling down to 298 K, 100 mL/min CO_2_ was fed to the sample. thermogravimetric and differential scanning calorimetry signals were simultaneously monitored throughout the process.

**The temperature‐programmed desorption (TPD) experiments**: The experiments were performed on a fixed‐bed packed with ∼0.5 g samples. The gas mixture of CO_2_/N_2_ was fed to the adsorber at a flow rate of 10 mL/min. After the fixed‐bed is saturated, the samples were purged by a flow of Ar at 30 mL/min for 20 min and then the samples were then heated from 298 to 573 K. The temperature‐programmed desorption (TPD) profiles of CO_2_ were recorded by mass spectrometry.

^**13**^ **C cross‐polarization/magic angle spinning nuclear magnetic resonance (CP/MAS NMR) measurements**: NMR tests were performed on a Bruker Avance III 400WB spectrometer at the resonance frequency of 100.6 MHz, with the contact time of 3 ms, the repetition time of 4 s, and the sample spinning rate of 10.0 kHz. Typically, the sample was pretreated under vacuum at 623 K for 10 h (in a home‐made high‐vacuum line) to remove trace adsorbates such as water. After cooling down at 298 K in vacuum, ^13^CO_2_ was introduced to the sample at ∼10 mbar. Then the sample was transferred into a rotor inside a glove box without contact with air for ^13^C CP/MAS NMR tests.

**In situ neutron powder diffraction (NPD)**: Structural determination of the binding positions of CO_2_ within Na−X were conducted on WISH, a long‐wavelength powder and single‐crystal neutron diffractometer at the ISIS neutron and muon facility at Rutherford Appleton Laboratory (UK). The instrument incorporates a solid methane moderator, providing a high flux of cold neutrons with a large bandwidth, which is transported to the sample via an elliptical guide. The divergence jaws of WISH allow tuning of the resolution according to the need of the experiment; in this case, it was setup in high‐resolution mode. The WISH detectors are 1 m long, 8 mm diameter pixelated ^3^He tubes positioned at 2.2 m from the sample and arranged on a cylindrical locus covering a 2 θ scattering angle of 10–170°. To reduce the background from the sample environment, WISH is equipped with an oscillating radial collimator that defines a cylinder of radius of approximately 22 mm diameter at 90° scattering. The sample of desolvated Na−X was loaded into a cylindrical vanadium sample container with an indium vacuum seal connected to a gas handling system. The sample was degassed at 1×10^−7^ mbar and at 383 K for 4 days with He flushing to remove any remaining trace guest water molecules. The sample was dosed with CO_2_ using the volumetric method at room temperature to ensure that the gas was well dispersed throughout the crystalline structure of Na−X. Data collection was performed at 10±0.2 K, controlled using a helium cryostat.

**High‐resolution synchrotron X‐ray powder diffraction (PXRD)**: High resolution powder X‐ray diffraction patterns of Ba−X were collected on the powder diffractometer [λ=0.826833(10) Å] at 298 K on beamline I11 (Diamond Light Source, UK). The sample was degassed at 1×10^−7^ mbar and at 473 K for 12 h to remove any guest water molecules. To prevent preferred orientations, transmission geometry was used with 0.7 mm capillary. Data were collected between 0 and 150° using a step size of 0.001° with five multi‐analysing crystal (MAC) detectors.

TOPAS 5 (A. A. Coelho, *J. Appl. Cryst*. **2018**, 51, 210) was used to perform Pawley and Rietveld refinement on both NPD and PXRD patterns. Background, cell parameters and peak profile were first refined under the Pawley refinement and then transferred to the Rietveld refinement. The refined structural parameters include the fractional coordinates (*x*, *y*, *z*) and isotropic displacement factors for all atoms, and the site occupancy factors (SOF) for guest molecules. Rigid body refinement was applied to the guest molecules in the pore. The quality of the Rietveld refinements was assured with low weighted profile factors and well‐fitted patterns with reasonable isotropic displacement factors within experimental errors.

**Calculation of Henry constant and Q_st_**: Henry's constant from adsorption isotherms at the zero surface coverage is determined as the limiting slop of the adsorption isotherm:

(2) $$K_{H}^{0}=\underset{P\to 0}{lim}\;\left(\frac{n}{P}\right)=\underset{P\to 0}{lim}\;\left(\frac{dn}{dP}\right)$$

where $K_{H}^{0}$ is Henry's law constant (mol kg^−1^ kPa^−1^), P is pressure (kPa) and *n* is gas loading (mol kg^−1^). Low‐pressure CO_2_ isotherms on X are non‐linear, and the virial plot method was employed for the calculation of Henry's law constants. Virial plots are generated from the equation:

(3) $$\frac{P}{n}=\frac{1}{K_{H}^{0}}\exp\left(2A_{1}n+\frac{3}{2}A_{1}n^{2}+\frac{4}{3}A_{2}n^{3}+\cdots \right)$$

where *A_1_*, *A_2_*, *A_3_* are the virial coefficients. The temperature dependence of Henry's constant yields the isosteric heat at the limit of zero coverage, $Q_{st}^{0}$ (kJ mol^−1^)

(4) $$Q_{st}^{0}=-R\left[\frac{\partial \ln K_{H}^{0}}{\partial \left(1/T\right)}\right]$$

## Conflict of interest

The authors declare no conflict of interest.

1

## Supporting information

As a service to our authors and readers, this journal provides supporting information supplied by the authors. Such materials are peer reviewed and may be re‐organized for online delivery, but are not copy‐edited or typeset. Technical support issues arising from supporting information (other than missing files) should be addressed to the authors.

Supporting Information

## Data Availability

The data that support the findings of this study are available in the supplementary material of this article.
