# Supplementary material for: Regulating Extra‐Framework Cations in Faujasite Zeolites for Capture of Trace Carbon Dioxide
Source: Chemistry. 2022 Jul 13;28(50):e202201659. doi: 10.1002/chem.202201659 (PMC9545100; doi:10.1002/chem.202201659)
Supplement: Supplementary file 1 — Supporting Information [file CHEM-28-0-s001.pdf]

Chemistry–A European Journal

Supporting Information

Regulating Extra-Framework Cations in Faujasite Zeolites for Capture of Trace Carbon Dioxide

Shanshan Liu, Yinlin Chen, Bin Yue, Chang Wang, Bin Qin, Yuchao Chai, Guangjun Wu, Jiangnan Li, Xue Han, Ivan da-Silva, Pascal Manuel, Sarah J. Day, Stephen P. Thompson, Naijia Guan, Sihai Yang,* and Landong Li*

Table of Contents

Additional Discussion 4
Figures 5
Figure S1. XRD patterns of M-X zeolites. 5
Figure S2. Argon adsorption-desorption isotherms of M-X zeolites at 87 K. 5
Figure S3. CO_2 adsorption isotherms on M-X zeolites at 298 K. 6
Figure S4. Dynamic gas breakthrough tests of 5000 ppm CO_2 in synthetic air on M-X zeolites. Conditions: 0.5 g adsorbent, 298 K, 1 bar and total flow rate of 20 mL/min. 6
Figure S5. Dynamic gas breakthrough tests of 3000 ppm CO_2 in synthetic air on M-X zeolites. Conditions: 0.5 g adsorbent, 298 K, 1 bar and total flow rate of 20 mL/min. 7
Figure S6. Dynamic gas breakthrough tests of 1000 ppm CO_2 in synthetic air on M-X zeolites. Conditions: 0.5 g adsorbent, 298 K, 1 bar and total flow rate of 20 mL/min. 7
Figure S7. CO_2 and N_2 adsorption isotherms on Li-X, and the IAST selectivity for CO_2/N_2 (1/99) at 298 K. 8
Figure S8. CO_2 and N_2 adsorption isotherms on Na-X, and the IAST selectivity for CO_2/N_2 (1/99) at 298 K. 8
Figure S9. CO_2, N_2, CO, H_2 and CH_4 adsorption isotherms on K-X, and the IAST selectivity for CO_2/N_2 (CH_4, CO, H_2) (1/99) at 298 K. 9
Figure S10. CO_2 and N_2 adsorption isotherms on Cs-X, and the IAST selectivity for CO_2/N_2 (1/99) at 298 K. 9
Figure S11. CO_2 and N_2 adsorption isotherms on Mg-X, and the IAST selectivity for CO_2/N_2 (1/99) at 298 K. 10
Figure S12. CO_2 and N_2 adsorption isotherms on Ca-X, and the IAST selectivity for CO_2/N_2 (1/99) at 298 K. 10
Figure S13. Dynamic gas breakthrough tests of 10000 ppm CO_2 in synthetic air on Ba-X zeolites. Conditions: 0.5 g adsorbent, 298-323 K, 1 bar and total flow rate of 20 mL/min. 11
Figure S14. Dynamic gas breakthrough tests of 400 ppm CO_2 in synthetic air on Ba-X. Conditions: 0.5 g adsorbent, 298 K, 1 bar and total flow rate of 40 mL/min. 11
Figure S15. Dynamic gas breakthrough tests of 10000 ppm CO_2 in synthetic air with 74% RH on M-X zeolites. Conditions: 0.5 g adsorbent, 298 K, 1 bar and total flow rate of 10 mL/min. 12
Figure S16. Dynamic gas breakthrough tests of 5000 ppm CO_2 in synthetic air with 74% RH on M-X zeolites. Conditions: 0.5 g adsorbent, 298 K, 1 bar and total flow rate of 20 mL/min. 12
Figure S17. Dynamic gas breakthrough tests of 3000 ppm CO_2 in synthetic air with 74% RH on M-X zeolites. Conditions: 0.5 g adsorbent, 298 K, 1 bar and total flow rate of 20 mL/min. 13
Figure S18. Dynamic gas breakthrough tests of 1000 ppm CO_2 in synthetic air with 74% RH on M-X zeolites. Conditions: 0.5 g adsorbent, 298 K, 1 bar and total flow rate of 20 mL/min. 13
Figure S19. H_2O adsorption isotherms on Na-X and K-X at 298 K. 14
Figure S20. CO_2-TPD profiles of M-X zeolites. 14
Figure S21. Weight increase and heat flow of CO_2 adsorption on Li-X. 15
Figure S22. Weight increase and heat flow of CO_2 adsorption on Na-X. 15
Figure S23. Weight increase and heat flow of CO_2 adsorption on K-X. 16
Figure S24. Weight increase and heat flow of CO_2 adsorption on Cs-X. 16
Figure S25. Weight increase and heat flow of CO_2 adsorption on Mg-X. 17

Figure S26. Weight increase and heat flow of CO_2 adsorption on Ca-X. 17
Figure S27. Weight increase and heat flow of CO_2 adsorption on Ba-X. 18
Figure S28. Virial plots of CO_2 on Li-X at 273 K and 303 K. 18
Figure S29. Virial plots of CO_2 on Na-X at 273 K and 303 K. 19
Figure S30. Virial plots of CO_2 on K-X at 273 K and 303 K. 19
Figure S31. Virial plots of CO_2 on Mg-X at 273 K and 303 K. 20
Figure S32. Virial plots of CO_2 on Ca-X at 273 K and 303 K. 20
Figure S33. Virial plots of CO_2 on Ba-X at 273 K and 303 K. 21
Figure S34. XRD patterns of M-Y zeolites. 21
Figure S35. Argon adsorption-desorption isotherms of M-X zeolites at 87 K. 22
Figure S36. CO_2 adsorption isotherms of M-Y zeolites at 298 K. 22
Figure S37. Dynamic gas breakthrough tests of 10000 ppm CO_2 in synthetic air on M-Y zeolites. Conditions: 0.5 g adsorbent, 298 K, 1 bar and total flow rate of 20 mL/min. 23
Figure S38. Dynamic gas breakthrough tests of 5000 ppm CO_2 in synthetic air on M-Y zeolites. Conditions: 0.5 g adsorbent, 298 K, 1 bar and total flow rate of 20 mL/min. 23
Figure S39. Dynamic gas breakthrough tests of 3000 ppm CO_2 in synthetic air on M-Y zeolites. Conditions: 0.5 g adsorbent, 298 K, 1 bar and total flow rate of 20 mL/min. 24
Figure S40. Dynamic gas breakthrough tests of 1000 ppm CO_2 in synthetic air on M-Y zeolites. Conditions: 0.5 g adsorbent, 298 K, 1 bar and total flow rate of 20 mL/min. 24
Figure S41. Weight increase and heat flow of CO_2 adsorption on Li-Y. 25
Figure S42. Weight increase and heat flow of CO_2 adsorption on Na-Y. 25
Figure S43. Weight increase and heat flow of CO_2 adsorption on K-Y. 26
Figure S44. Weight increase and heat flow of CO_2 adsorption on Cs-Y. 26
Figure S45. Weight increase and heat flow of CO_2 adsorption on Mg-Y. 27
Figure S46. Weight increase and heat flow of CO_2 adsorption on Ca-Y. 27
Figure S47. Weight increase and heat flow of CO_2 adsorption on Ba-Y. 28
Figure S48. Correlation between the calculated mean charge on framework oxygen atom and the measured Q_{st} of CO_2 adsorption on M-Y zeolites. 28
Figure S49. Correlation between the measured Q_{st} of CO_2 adsorption and dynamic CO_2 (10,000 ppm) uptake in M-Y zeolites per 1000 m^2 microporous surface area. 29
Figure S50. Neutron diffraction patterns and Rietveld refinement for activated Na-X (banks 1 to 5 from top to bottom). 30
Figure S51. Neutron diffraction patterns and Rietveld refinement for CO_2-loaded Na-X (banks 1 to 5 from top to bottom). 31
Figure S52. Synchrotron X-ray diffraction patterns and Rietveld refinement for activated Ba-X (up: 2.5–50°, down: 20–50°). 32
Figure S53. Synchrotron X-ray diffraction patterns and Rietveld refinement for CO_2-loaded Ba-X (up: 2.5–50°, down: 20–50°). 33
Tables 34
Table S1. Textual properties of M-X zeolites. 34
Table S2. CO_2 adsorption data on M-X zeolites. 34
Table S3. CO_2 adsorption data of M-X zeolites on per standard volume. 35
Table S4. Comparison of adsorbents for DAC of CO_2 under ambient conditions. 36
Table S5. CO_2 adsorption data of M-X zeolites on per microporous surface area. 38

Table S6. Henry's law constants for CO_2 adsorption on M-X zeolites 38
Table S7. Textual properties of M-Y zeolites. 38
Table S8. Breakthrough data of M-Y zeolites in CO_2 capture 39
Table S9. Characteristics of oxygen charge on M-Y zeolites. 39
Table S10. Crystal Data and Details of the Structure Determination for M-X. 40
Reference 41

Additional Discussion

To verify the general applicability of the correlation between the basic strength of framework oxygen atoms, Q_{st} and dynamic CO_2 uptake, M-Y zeolites (FAU topology, Si/Al = 2.62) were investigated for CO_2 adsorption. The XRD patterns and argon adsorption-desorption isotherms of as-prepared M-Y zeolites are shown in **Figure S34** and **Figure S35**, respectively, and their texture properties are summarized in **Table S7**. The CO_2 adsorption isotherms of M-Y zeolites at 298 K are shown in **Figure S36**. In the low-pressure region (10 mbar), the static CO_2 uptake is observed as Ba-Y > Cs-Y > Ca-Y ≈ K-Y > Na-Y > Mg-Y ≈ Li-Y. The CO_2 breakthrough curves using synthetic air (CO_2 concentrations: 1,000-10,000 ppm) and the adsorption heat of CO_2 in M-Y zeolites are available in **Figure S37-40**. The dynamic uptake sequence at 10,000 ppm dry CO_2 is obtained as Ba-Y (0.99 mmol/g) > Cs-Y (0.83 mmol/g) > K-Y (0.40 mmol/g) > Ca-Y (0.26 mmol/g) > Na-Y (0.24 mmol/g) > Mg-Y (0.23 mmol/g) > Li-Y (0.16 mmol/g), and the uptake sequences at other concentrations are similar (**Table S8**). The Q_{st} sequence of CO_2 on M-Y zeolites is obtained as Cs-Y (51.1 kJ/mol) > Ba-Y (49.1 kJ/mol) > K-Y (37.5 kJ/mol) > Ca-Y (35.5 kJ/mol) > Mg-Y (29.2 kJ/mol) > Na-Y (26.0 kJ/mol) > Li-Y (23.9 kJ/mol) (**Figure S41-47**). The framework oxygen basicity of the M-Y zeolites, namely the mean charge on oxygen atom, is shown in **Table S8.** For M-Y zeolites, a near-linear correlation between the calculated mean charge on framework oxygen atom and the measured Q_{st} of CO_2 adsorption is obtained (**Figure S48**). The fewer ion-exchange sites of Y zeolites, in comparison with those of X zeolites, simplify the problem of extra-framework distribution and therefore a better correlation can be obtained. Meanwhile, a positive correlation between the measured Q_{st} of CO_2 adsorption and the dynamic CO_2 uptake in M-Y zeolites per microporous surface area can be obtained (**Figure S49**), despite that the relatively low dynamic CO_2 uptake derives some volatility to the data.

Figures

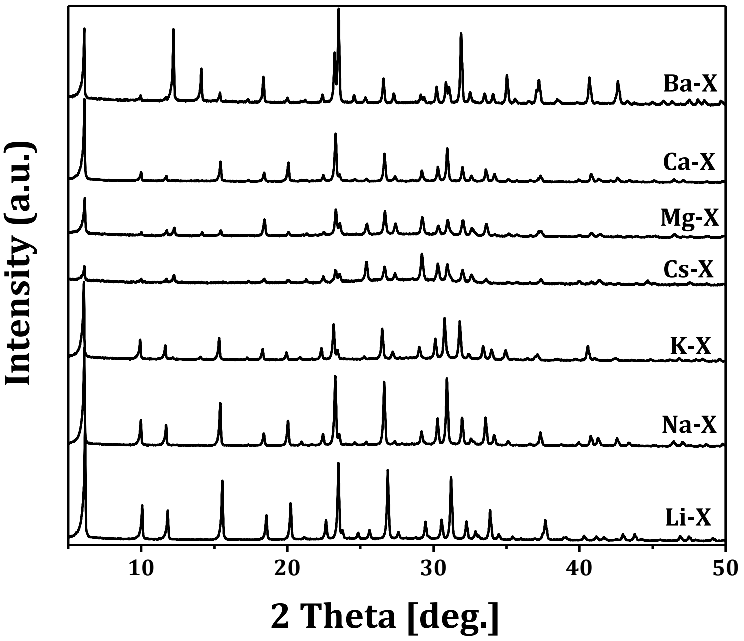

Figure S1. XRD patterns of M-X zeolites.

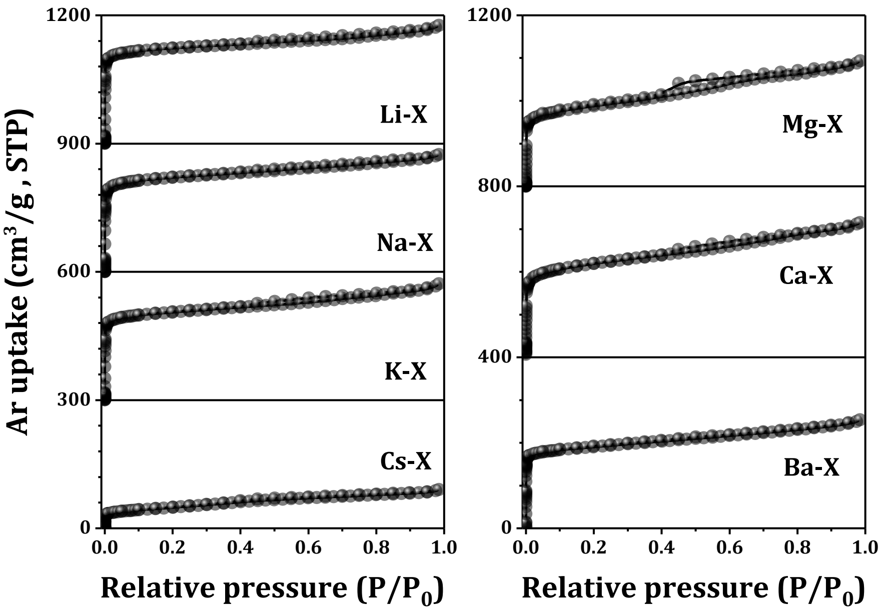

Figure S2. Argon adsorption-desorption isotherms of M-X zeolites at 87 K.

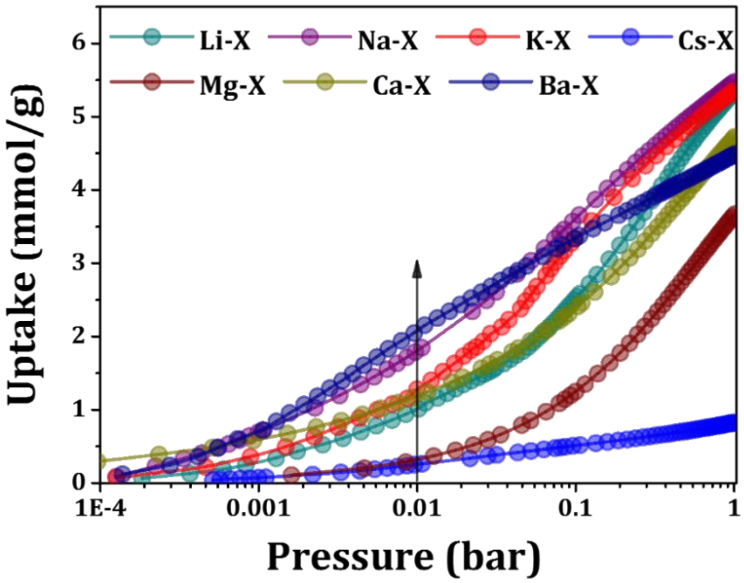

Figure S3. CO_2 adsorption isotherms on M-X zeolites at 298 K.

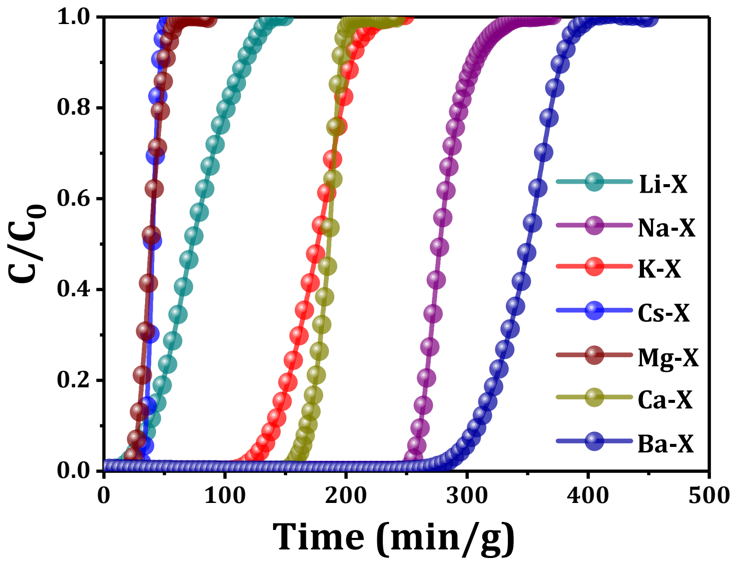

Figure S4. Dynamic gas breakthrough tests of 5000 ppm CO_2 in synthetic air on M-X zeolites. Conditions: 0.5 g adsorbent, 298 K, 1 bar and total flow rate of 20 mL/min.

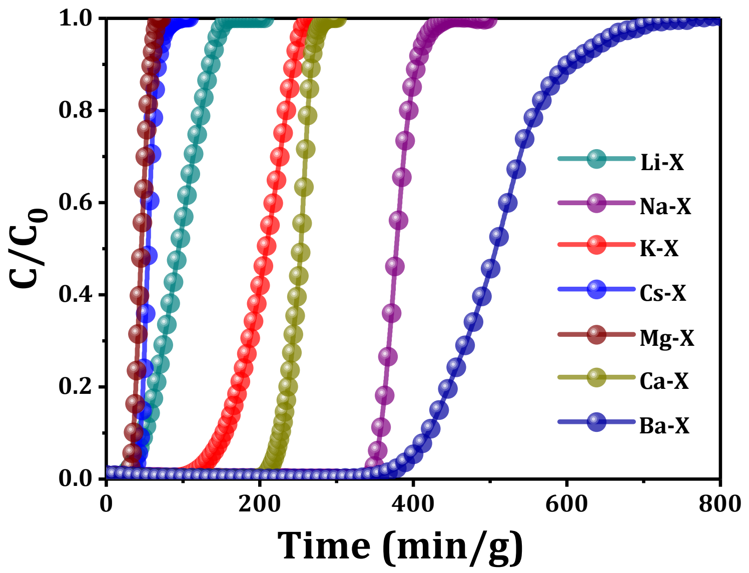

Figure S5. Dynamic gas breakthrough tests of 3000 ppm CO_2 in synthetic air on M-X zeolites. Conditions: 0.5 g adsorbent, 298 K, 1 bar and total flow rate of 20 mL/min.

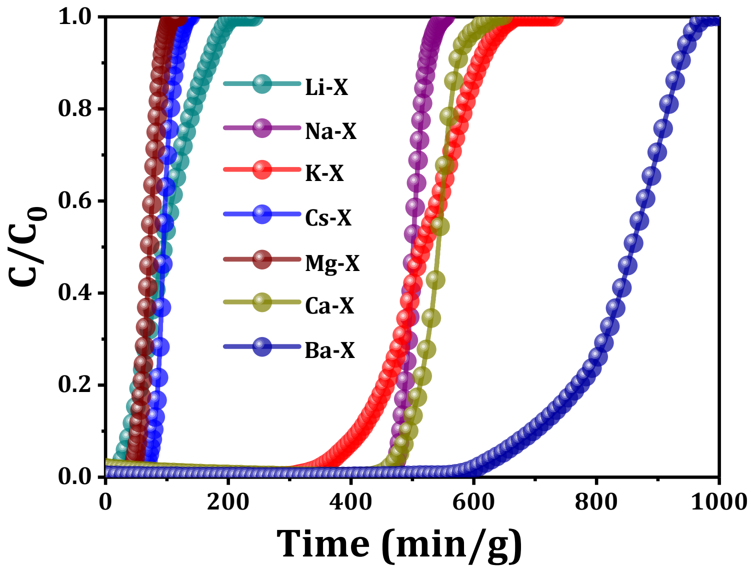

Figure S6. Dynamic gas breakthrough tests of 1000 ppm CO_2 in synthetic air on M-X zeolites. Conditions: 0.5 g adsorbent, 298 K, 1 bar and total flow rate of 20 mL/min.

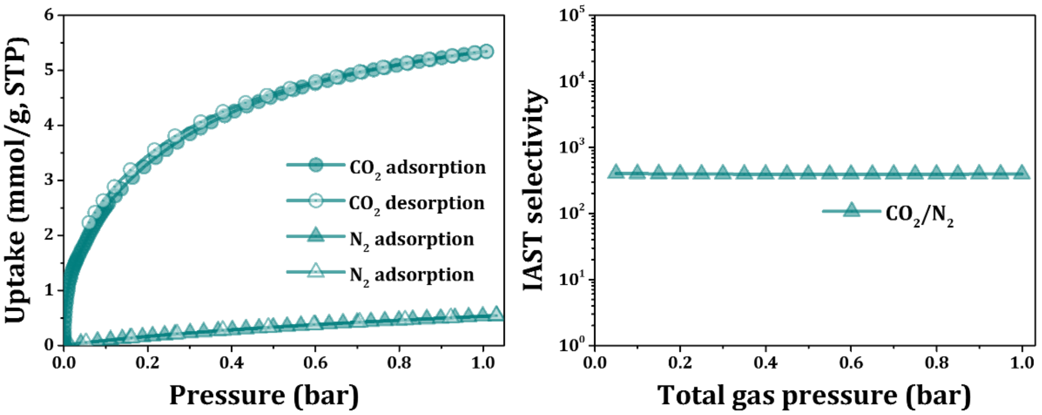

Figure S7. CO_2 and N_2 adsorption isotherms on Li-X, and the IAST selectivity for CO_2/N_2 (1/99) at 298 K.

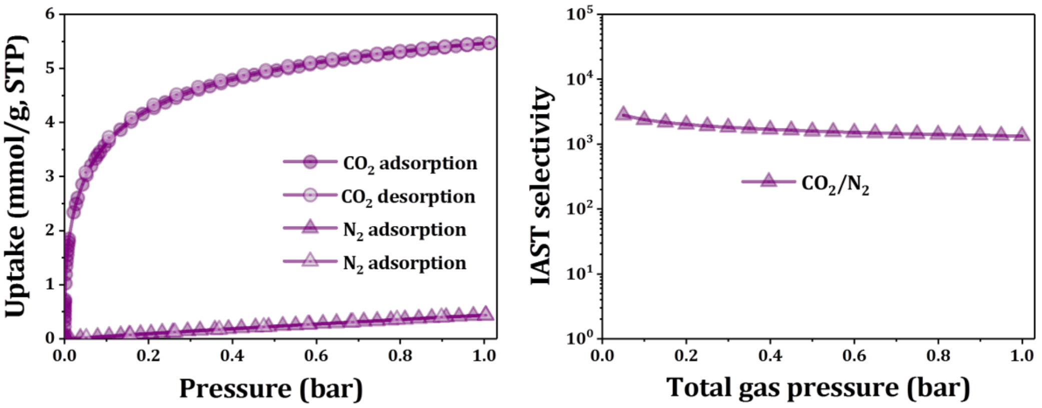

Figure S8. CO_2 and N_2 adsorption isotherms on Na-X, and the IAST selectivity for CO_2/N_2 (1/99) at 298 K.

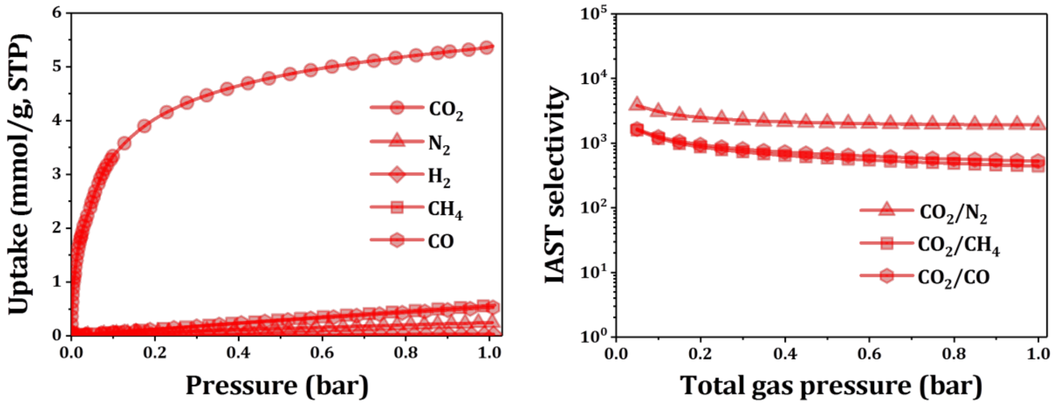

Figure S9. CO_2, N_2, CO, H_2 and CH_4 adsorption isotherms on K-X, and the IAST selectivity for CO_2/N_2 (CH_4, CO, H_2) (1/99) at 298 K.

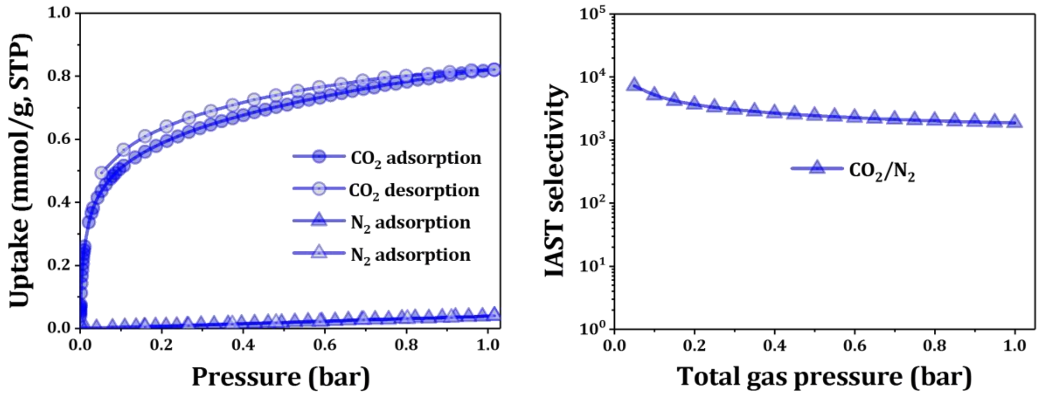

Figure S10. CO_2 and N_2 adsorption isotherms on Cs-X, and the IAST selectivity for CO_2/N_2 (1/99) at 298 K.

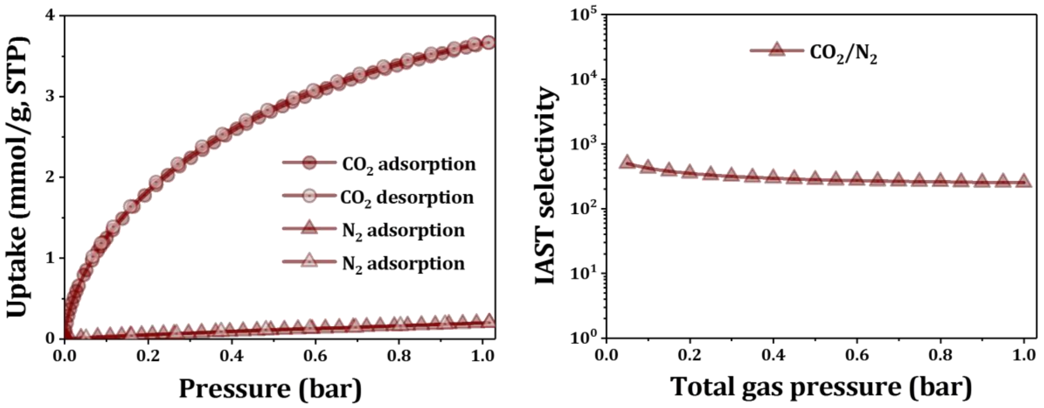

Figure S11. CO_2 and N_2 adsorption isotherms on Mg-X, and the IAST selectivity for CO_2/N_2 (1/99) at 298 K.

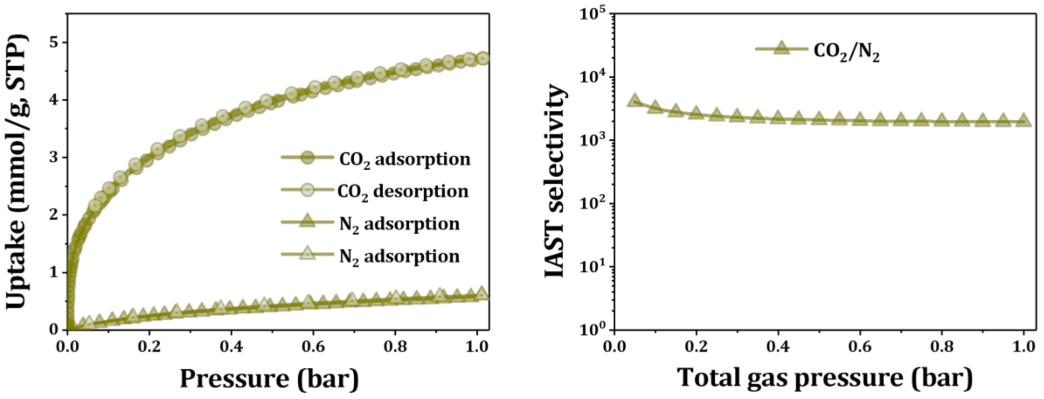

Figure S12. CO_2 and N_2 adsorption isotherms on Ca-X, and the IAST selectivity for CO_2/N_2 (1/99) at 298 K.

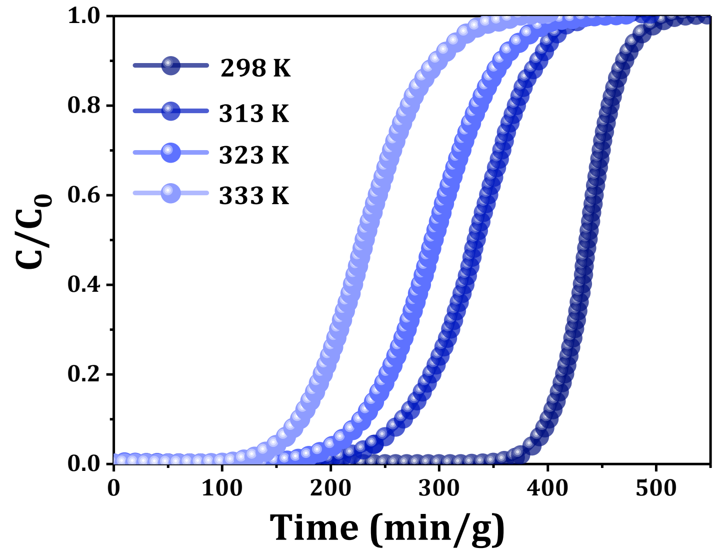

Figure S13. Dynamic gas breakthrough tests of 10000 ppm CO_2 in synthetic air on Ba-X zeolites. Conditions: 0.5 g adsorbent, 298-323 K, 1 bar and total flow rate of 20 mL/min.

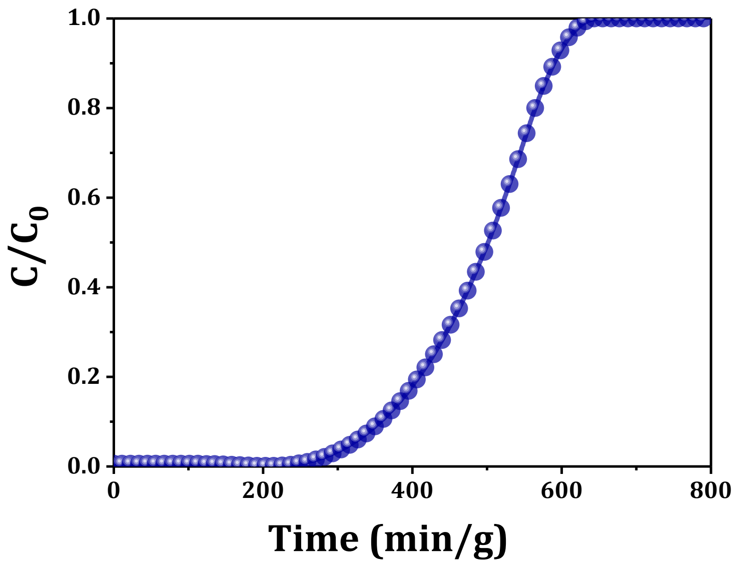

Figure S14. Dynamic gas breakthrough tests of 400 ppm CO_2 in synthetic air on Ba-X. Conditions: 0.5 g adsorbent, 298 K, 1 bar and total flow rate of 40 mL/min.

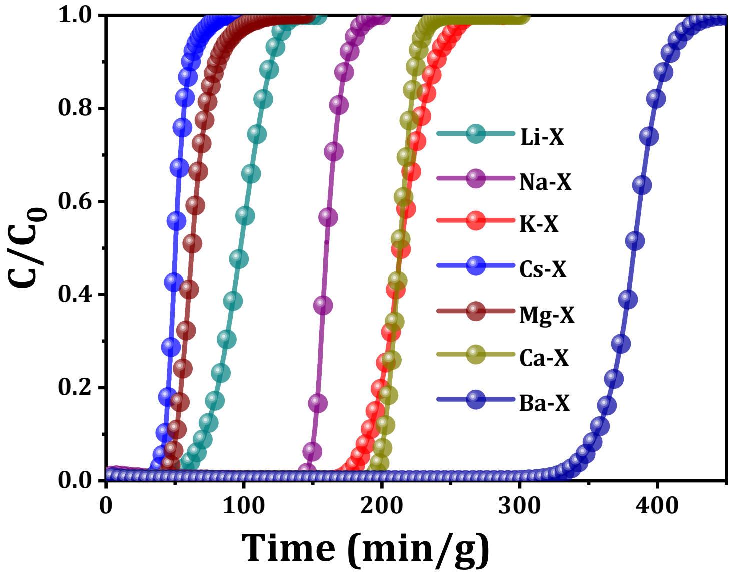

Figure S15. Dynamic gas breakthrough tests of 10000 ppm CO_2 in synthetic air with 74% RH on M-X zeolites. Conditions: 0.5 g adsorbent, 298 K, 1 bar and total flow rate of 10 mL/min.

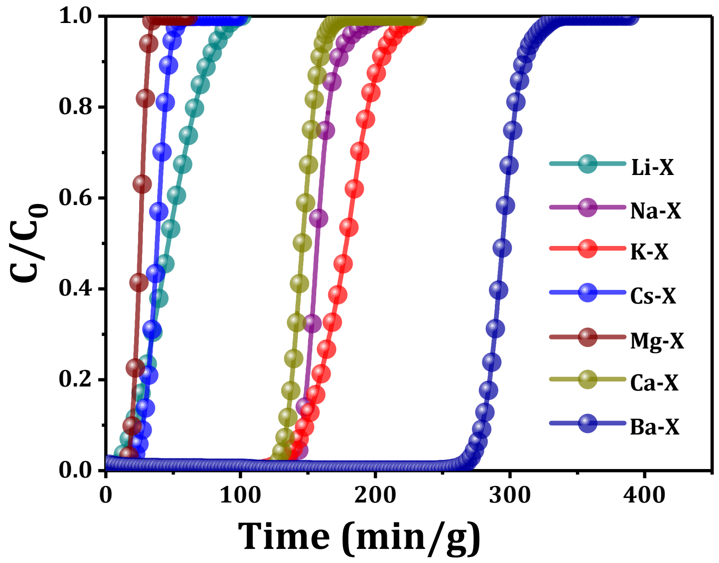

Figure S16. Dynamic gas breakthrough tests of 5000 ppm CO_2 in synthetic air with 74% RH on M-X zeolites. Conditions: 0.5 g adsorbent, 298 K, 1 bar and total flow rate of 20 mL/min.

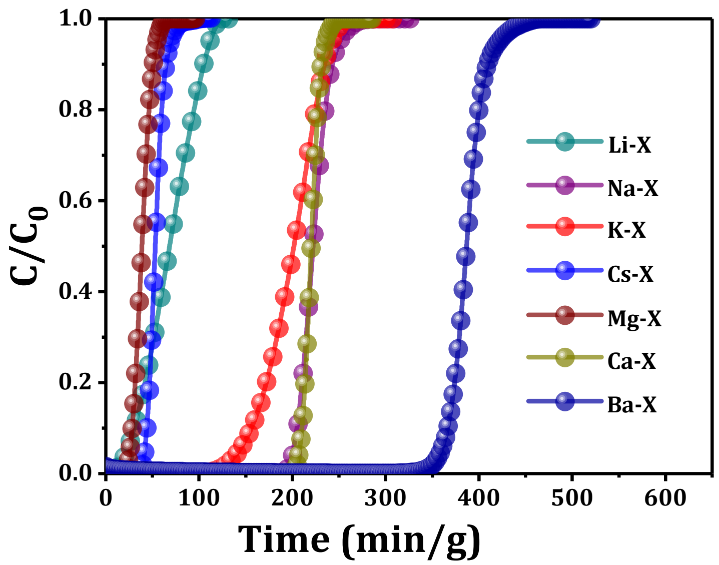

Figure S17. Dynamic gas breakthrough tests of 3000 ppm CO_2 in synthetic air with 74% RH on M-X zeolites. Conditions: 0.5 g adsorbent, 298 K, 1 bar and total flow rate of 20 mL/min.

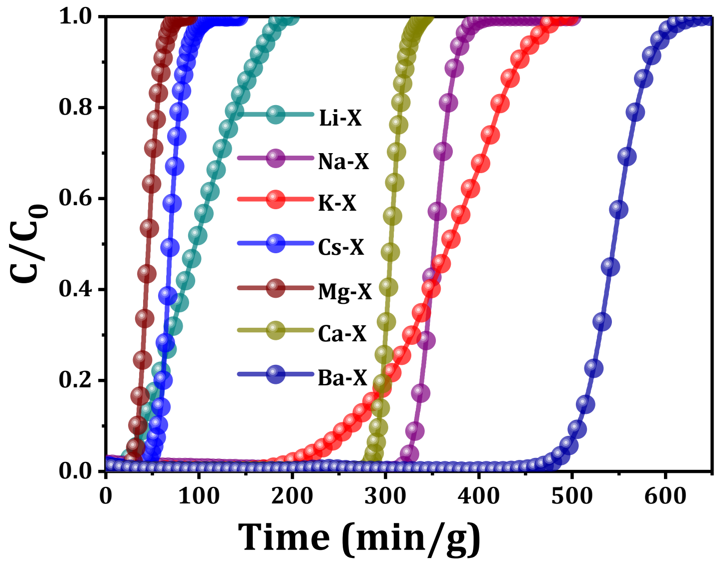

Figure S18. Dynamic gas breakthrough tests of 1000 ppm CO_2 in synthetic air with 74% RH on M-X zeolites. Conditions: 0.5 g adsorbent, 298 K, 1 bar and total flow rate of 20 mL/min.

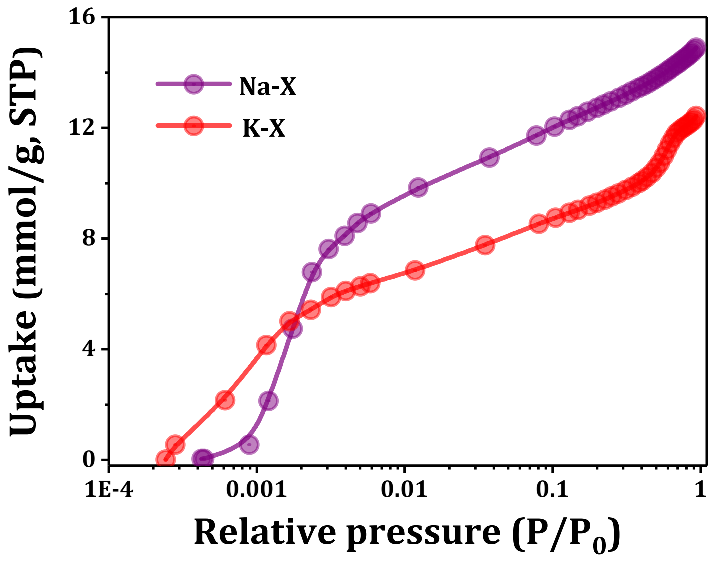

Figure S19. H_2O adsorption isotherms on Na-X and K-X at 298 K.

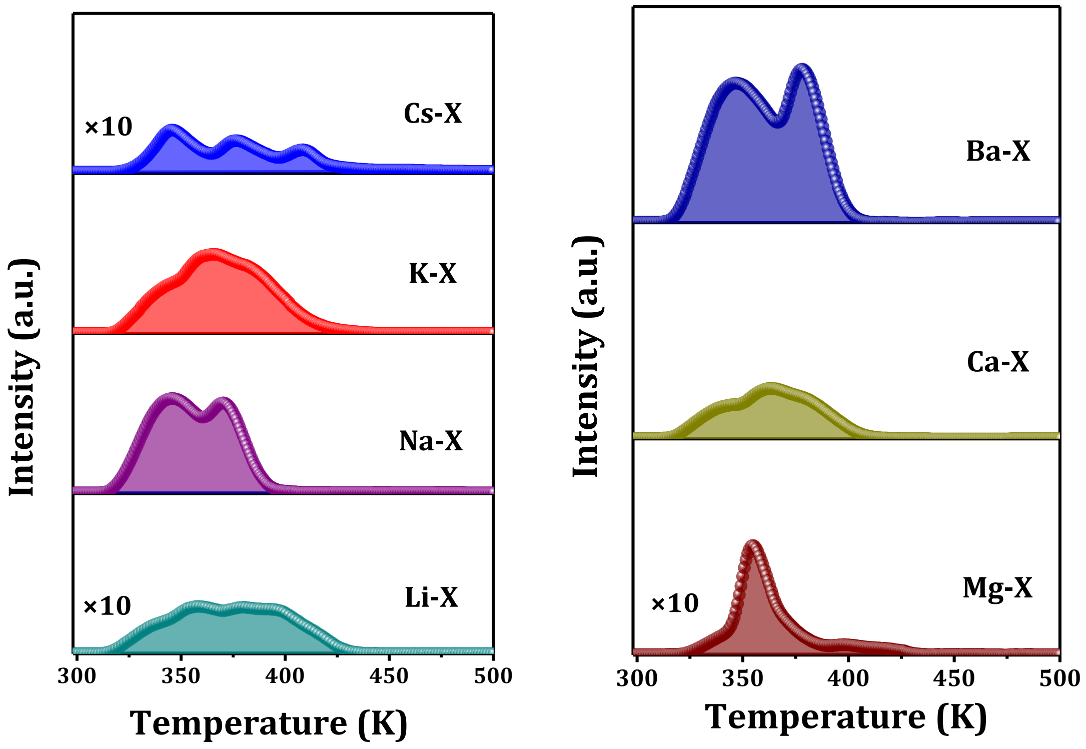

Figure S20. CO_2-TPD profiles of M-X zeolites.

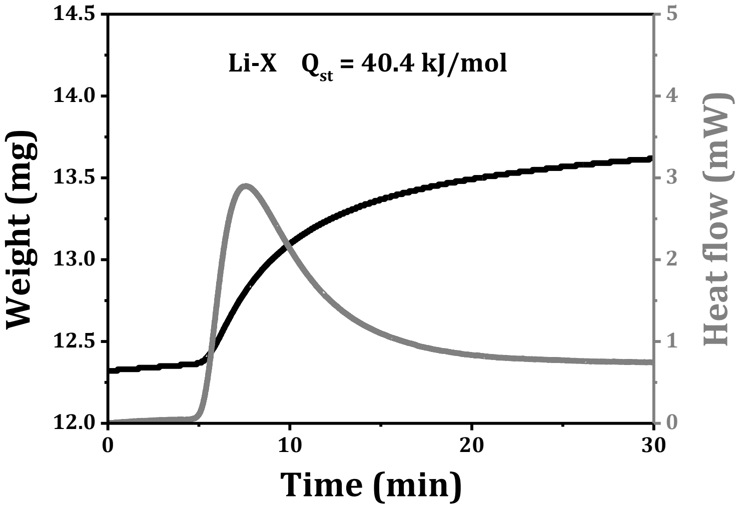

Figure S21. Weight increase and heat flow of CO_2 adsorption on Li-X.

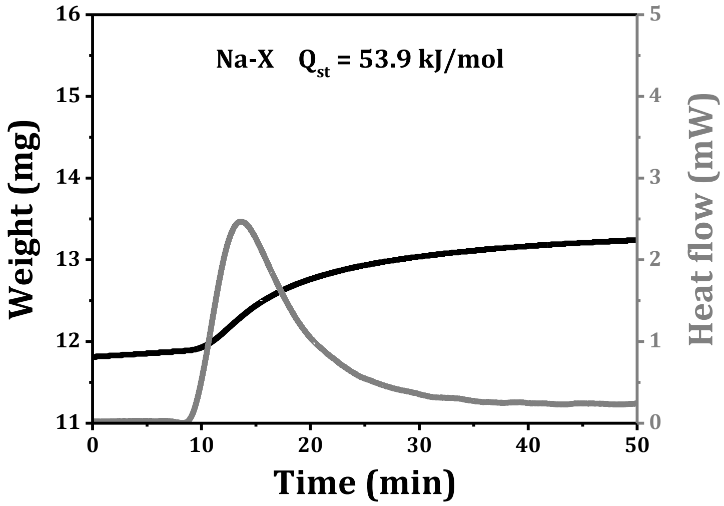

Figure S22. Weight increase and heat flow of CO_2 adsorption on Na-X.

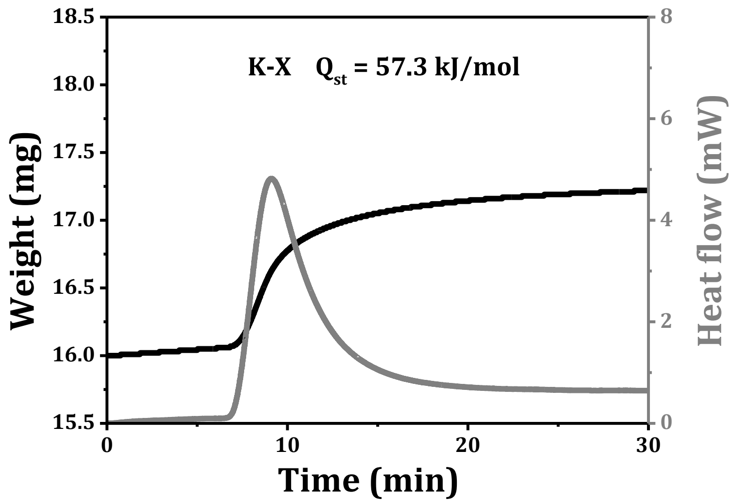

Figure S23. Weight increase and heat flow of CO_2 adsorption on K-X.

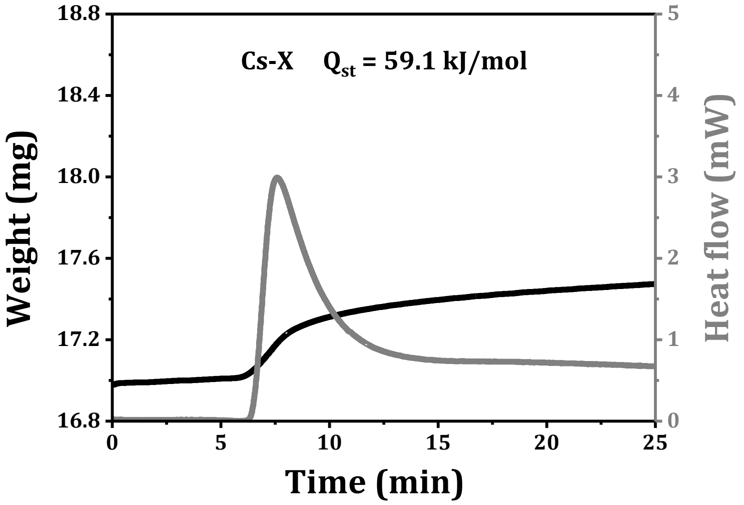

Figure S24. Weight increase and heat flow of CO_2 adsorption on Cs-X.

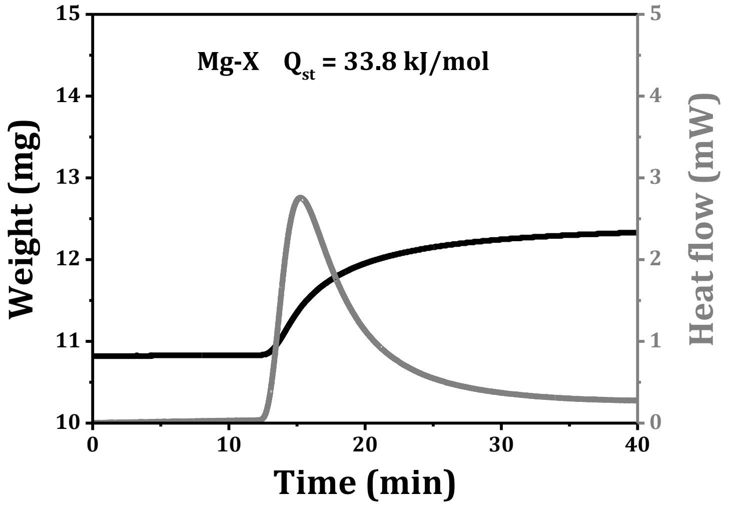

Figure S25. Weight increase and heat flow of CO_2 adsorption on Mg-X.

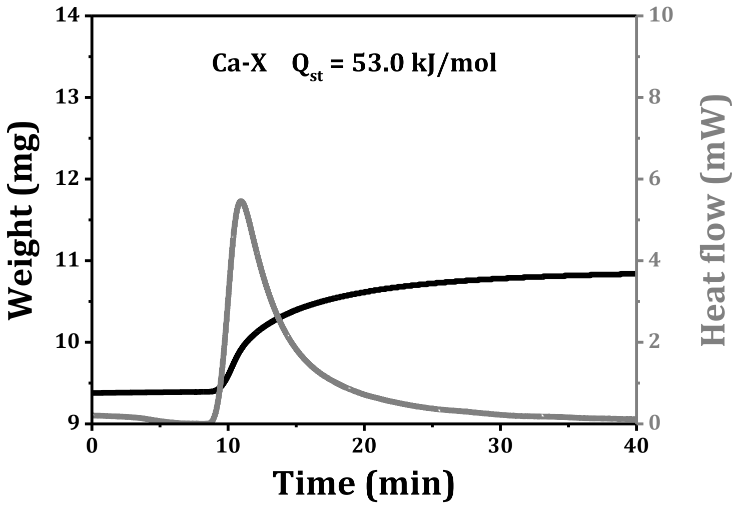

Figure S26. Weight increase and heat flow of CO_2 adsorption on Ca-X.

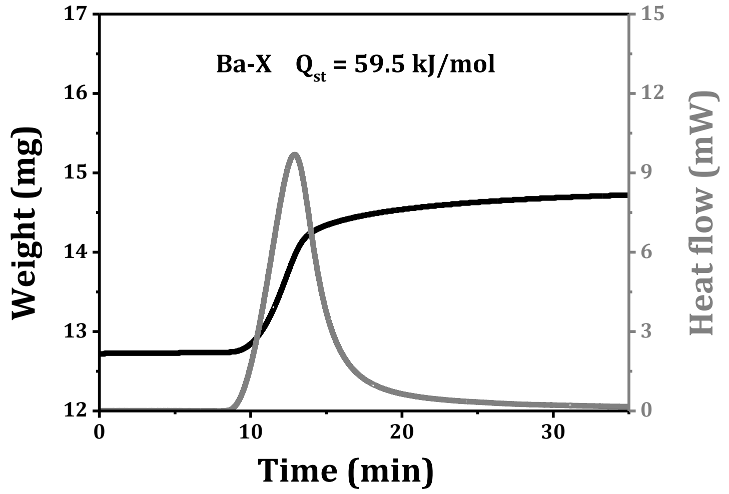

Figure S27. Weight increase and heat flow of CO_2 adsorption on Ba-X.

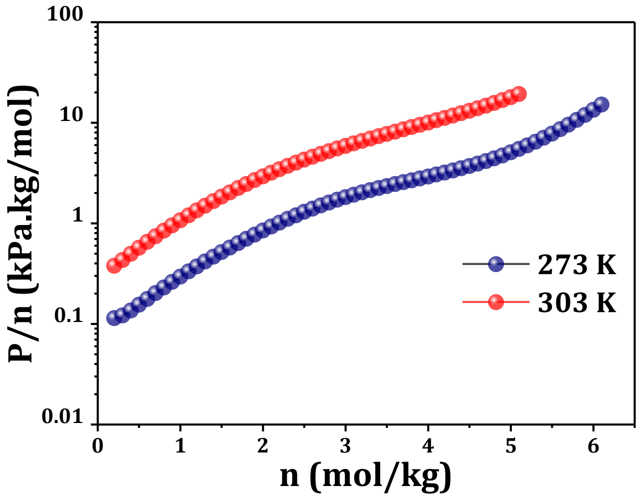

Figure S28. Virial plots of CO_2 on Li-X at 273 K and 303 K.

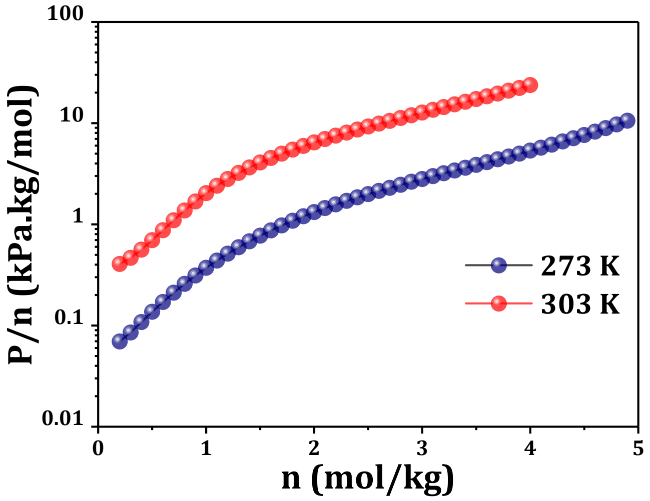

Figure S29. Virial plots of CO_2 on Na-X at 273 K and 303 K.

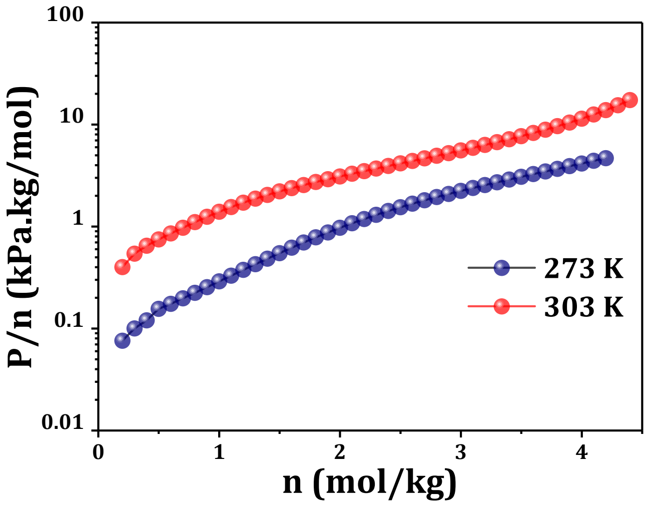

Figure S30. Virial plots of CO_2 on K-X at 273 K and 303 K.

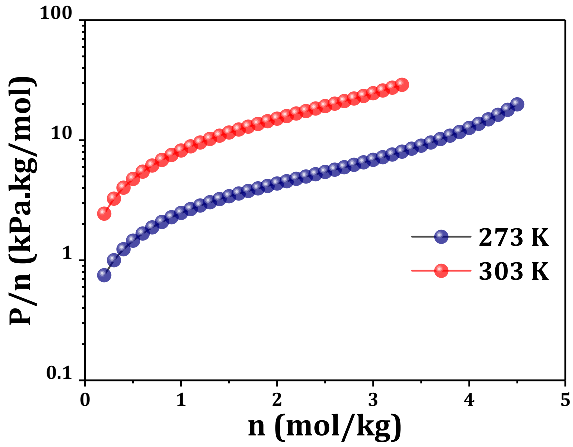

Figure S31. Virial plots of CO_2 on Mg-X at 273 K and 303 K.

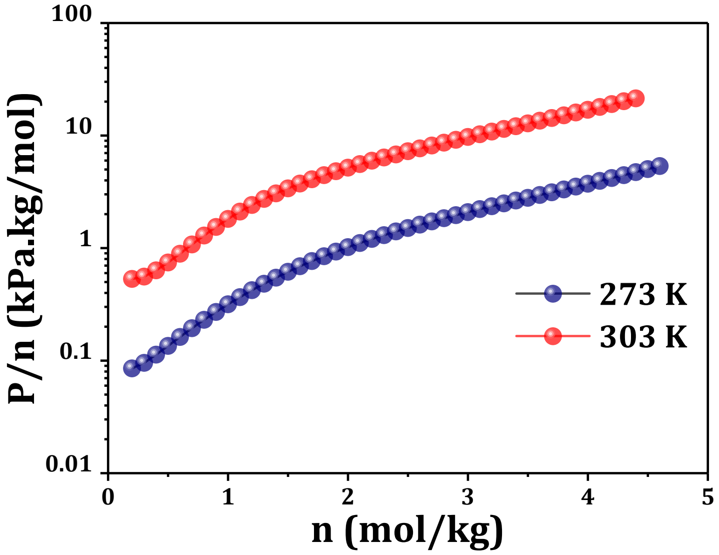

Figure S32. Virial plots of CO_2 on Ca-X at 273 K and 303 K.

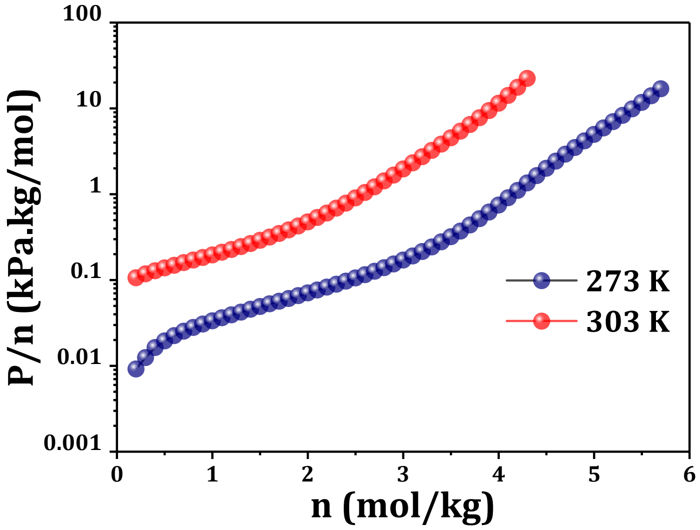

Figure S33. Virial plots of CO_2 on Ba-X at 273 K and 303 K.

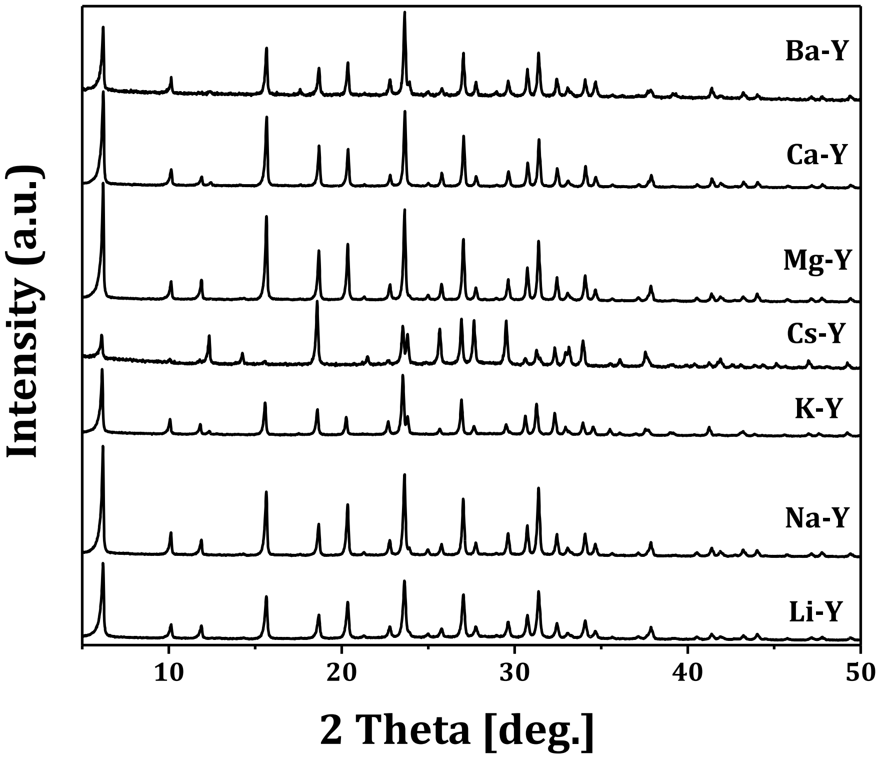

Figure S34. XRD patterns of M-Y zeolites.

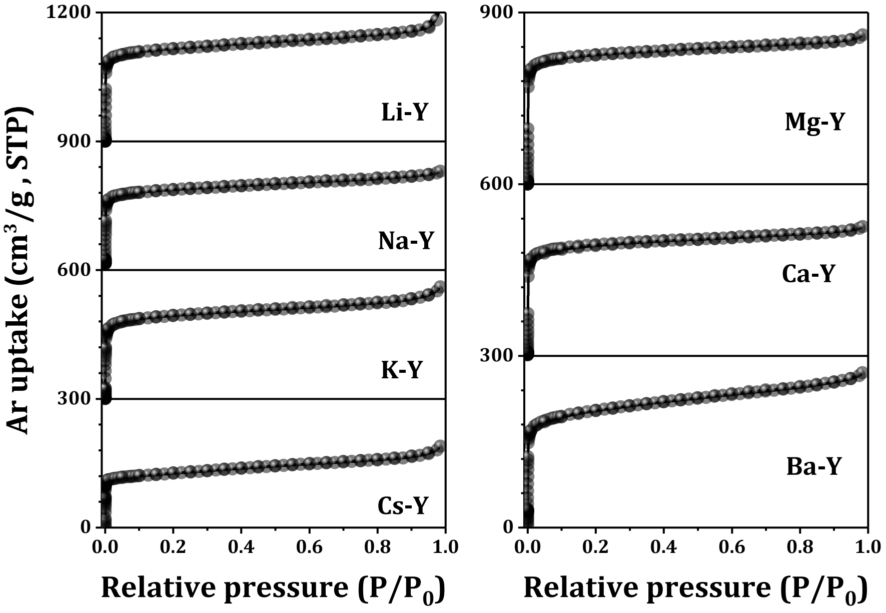

Figure S35. Argon adsorption-desorption isotherms of M-X zeolites at 87 K.

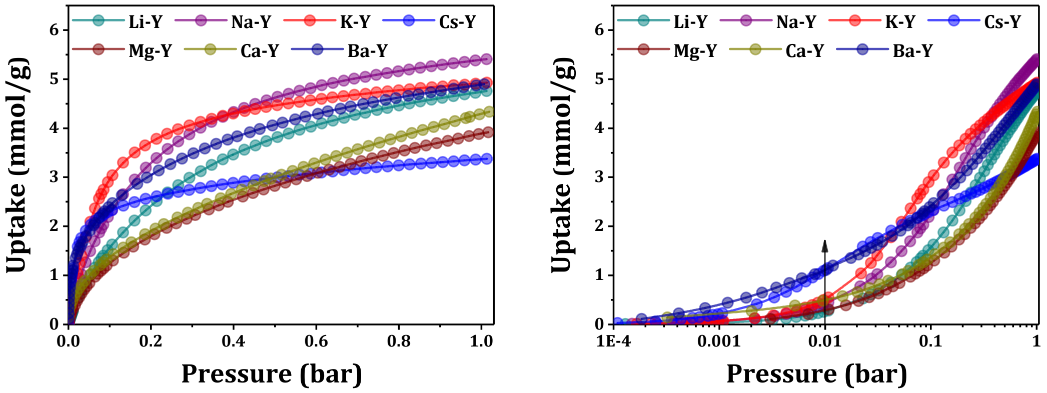

Figure S36. CO_2 adsorption isotherms of M-Y zeolites at 298 K.

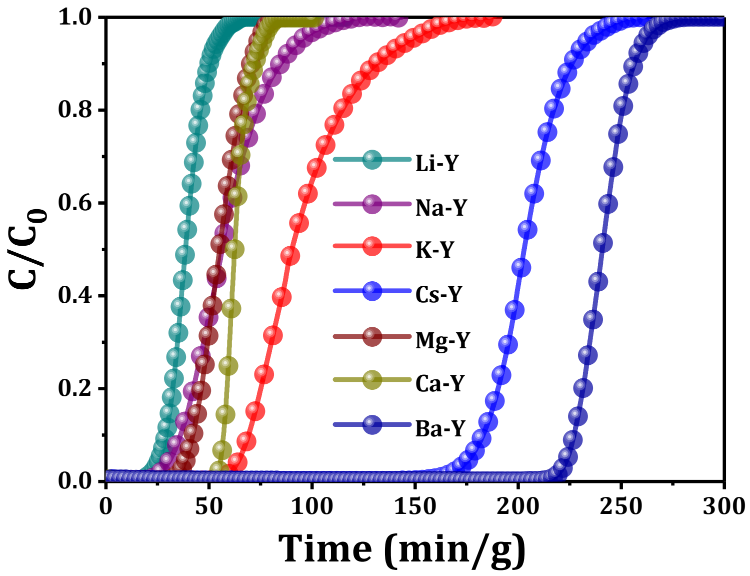

Figure S37. Dynamic gas breakthrough tests of 10000 ppm CO_2 in synthetic air on M-Y zeolites. Conditions: 0.5 g adsorbent, 298 K, 1 bar and total flow rate of 20 mL/min.

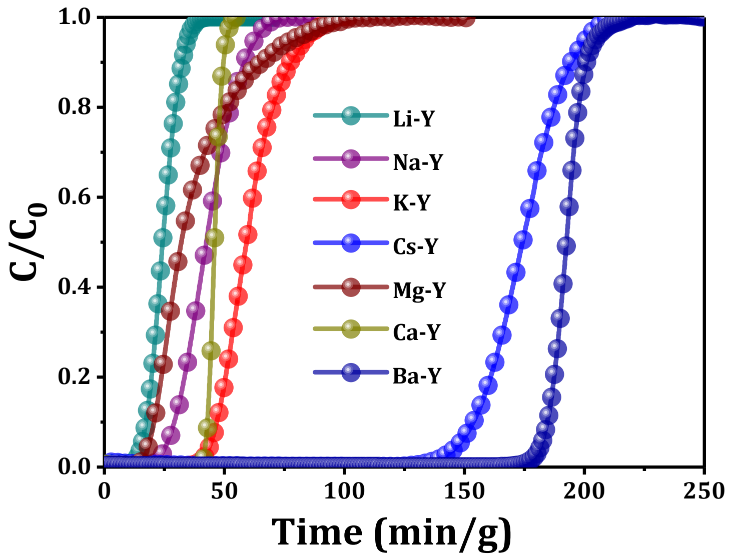

Figure S38. Dynamic gas breakthrough tests of 5000 ppm CO_2 in synthetic air on M-Y zeolites. Conditions: 0.5 g adsorbent, 298 K, 1 bar and total flow rate of 20 mL/min.

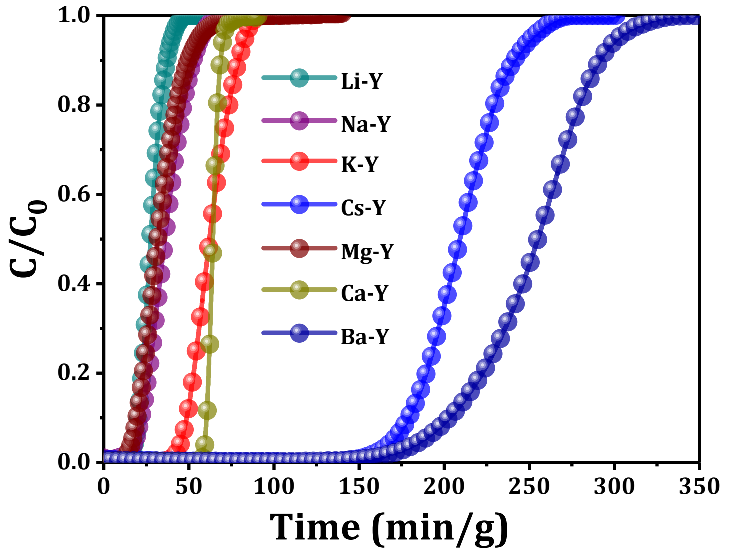

Figure S39. Dynamic gas breakthrough tests of 3000 ppm CO_2 in synthetic air on M-Y zeolites. Conditions: 0.5 g adsorbent, 298 K, 1 bar and total flow rate of 20 mL/min.

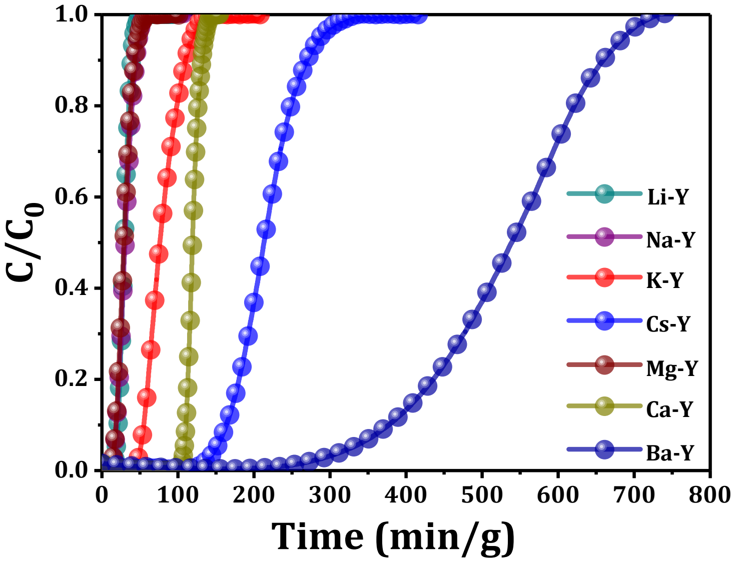

Figure S40. Dynamic gas breakthrough tests of 1000 ppm CO_2 in synthetic air on M-Y zeolites. Conditions: 0.5 g adsorbent, 298 K, 1 bar and total flow rate of 20 mL/min.

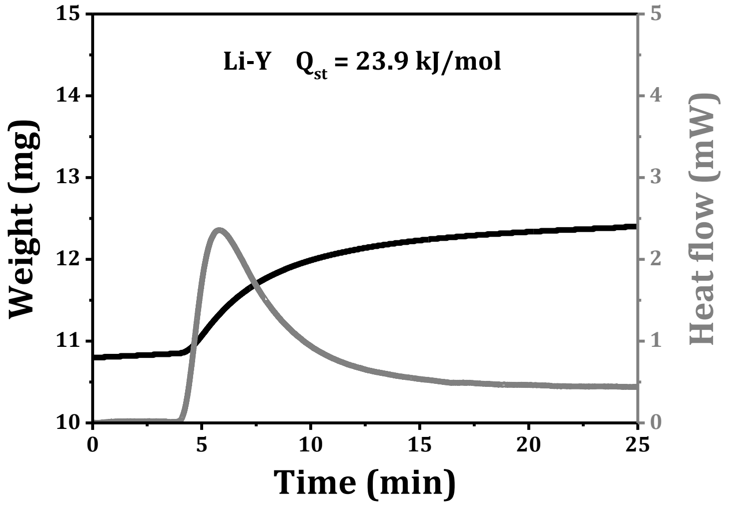

Figure S41. Weight increase and heat flow of CO_2 adsorption on Li-Y.

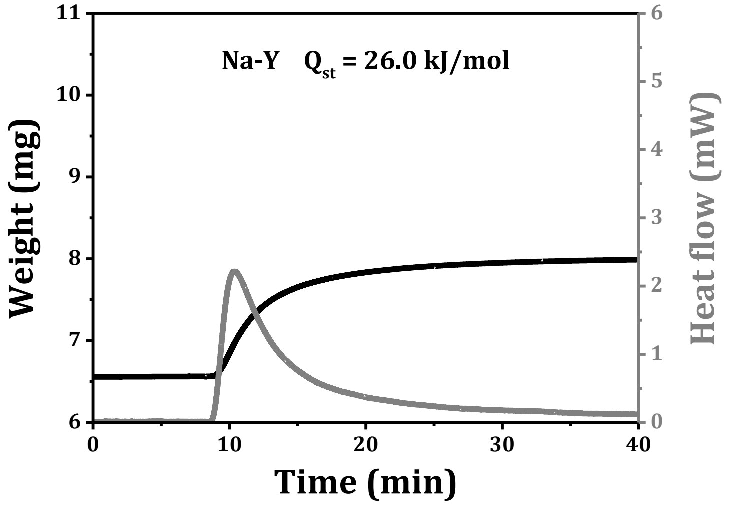

Figure S42. Weight increase and heat flow of CO_2 adsorption on Na-Y.

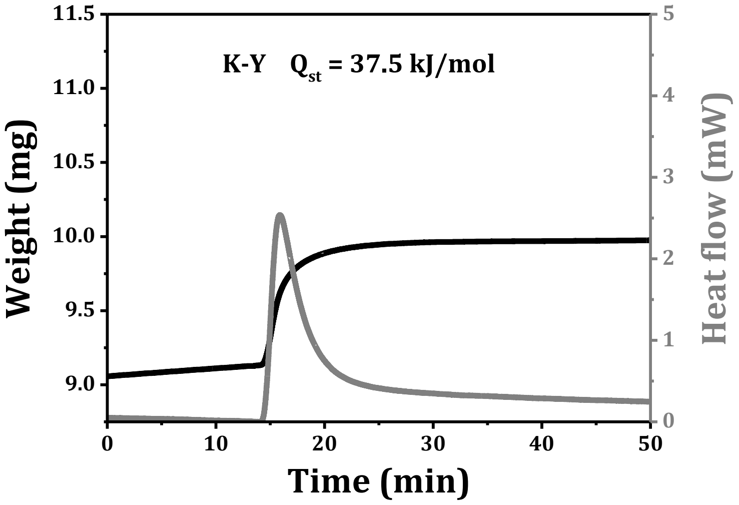

Figure S43. Weight increase and heat flow of CO_2 adsorption on K-Y.

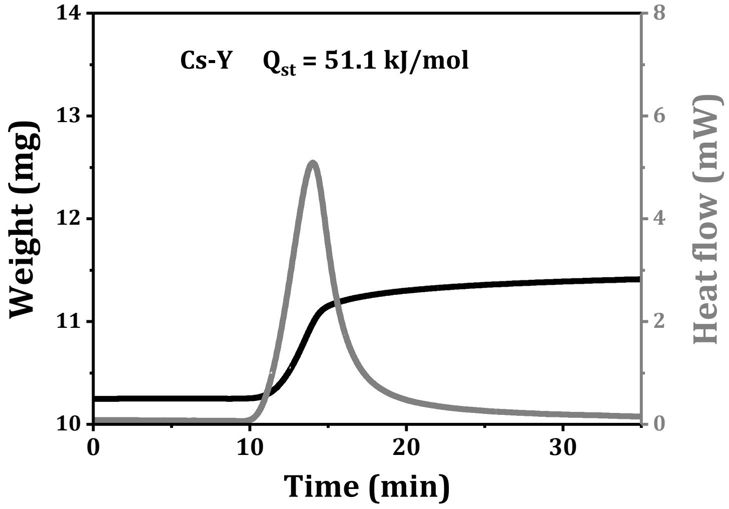

Figure S44. Weight increase and heat flow of CO_2 adsorption on Cs-Y.

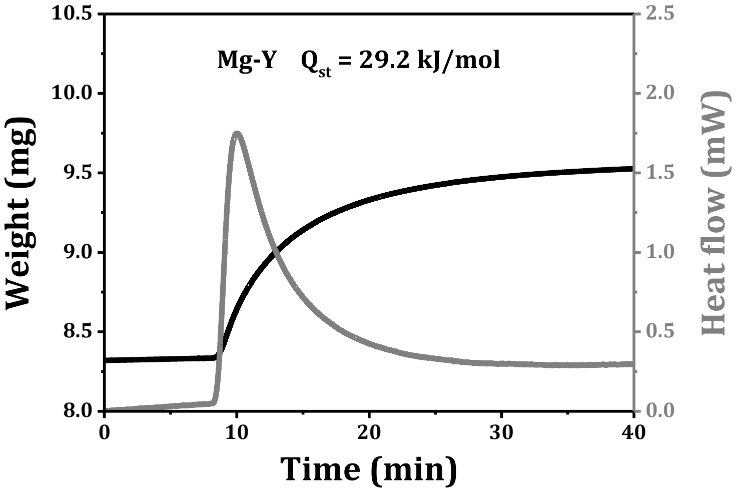

Figure S45. Weight increase and heat flow of CO_2 adsorption on Mg-Y.

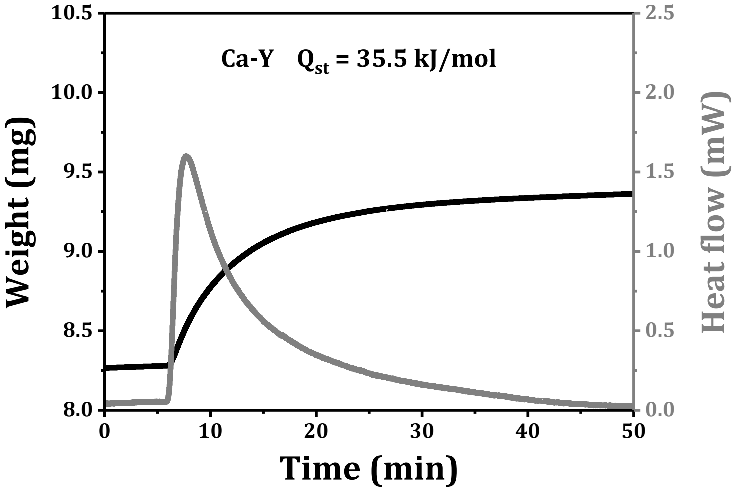

Figure S46. Weight increase and heat flow of CO_2 adsorption on Ca-Y.

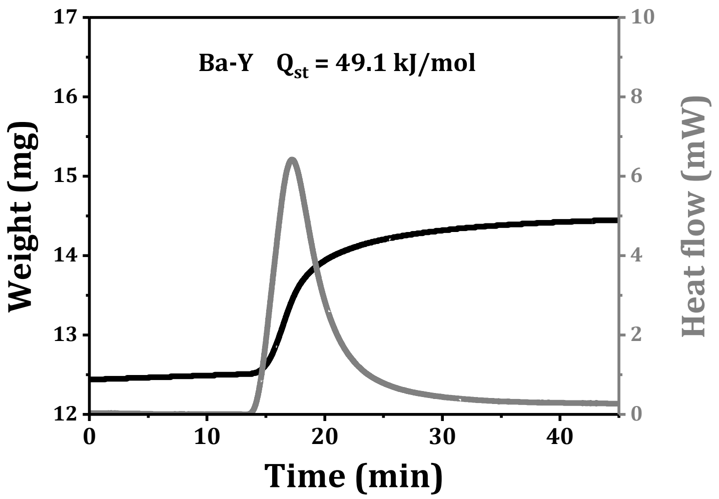

Figure S47. Weight increase and heat flow of CO_2 adsorption on Ba-Y.

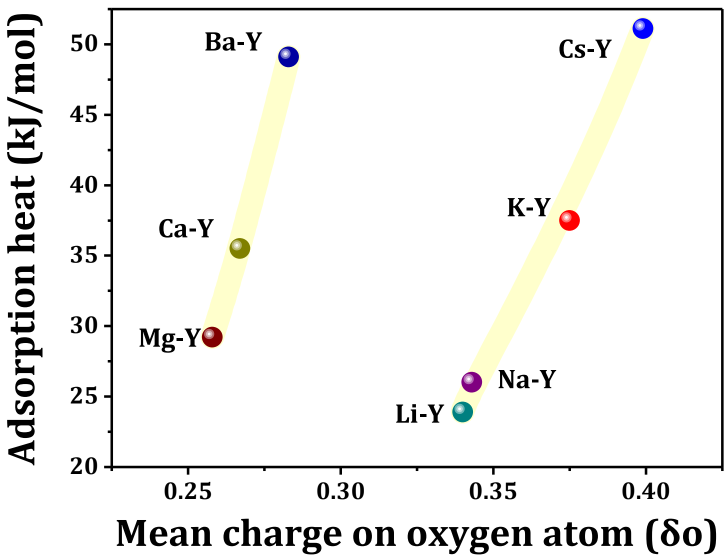

Figure S48. Correlation between the calculated mean charge on framework oxygen atom and the measured Q_{st} of CO_2 adsorption on M-Y zeolites

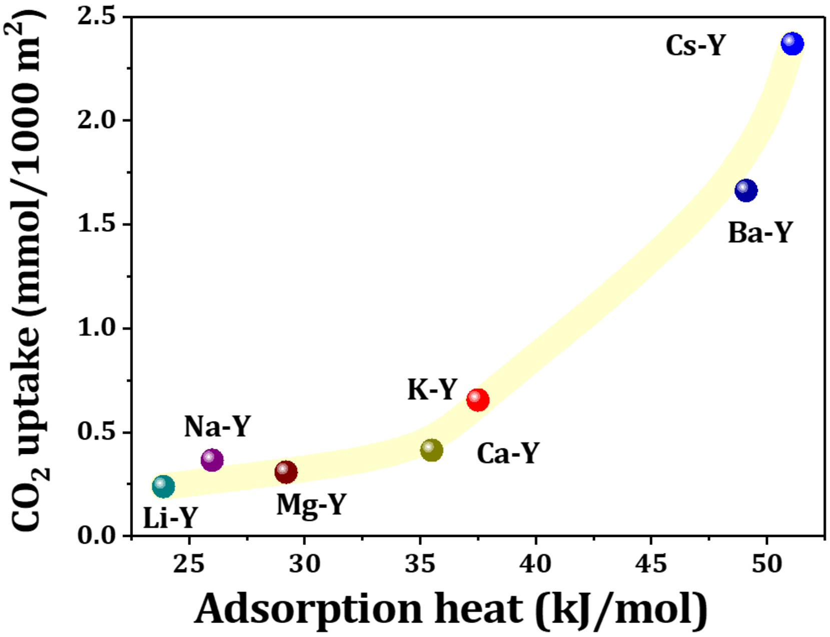

Figure S49. Correlation between the measured Q_{st} of CO_2 adsorption and dynamic CO_2 (10,000 ppm) uptake in M-Y zeolites per 1000 m^2 microporous surface area.

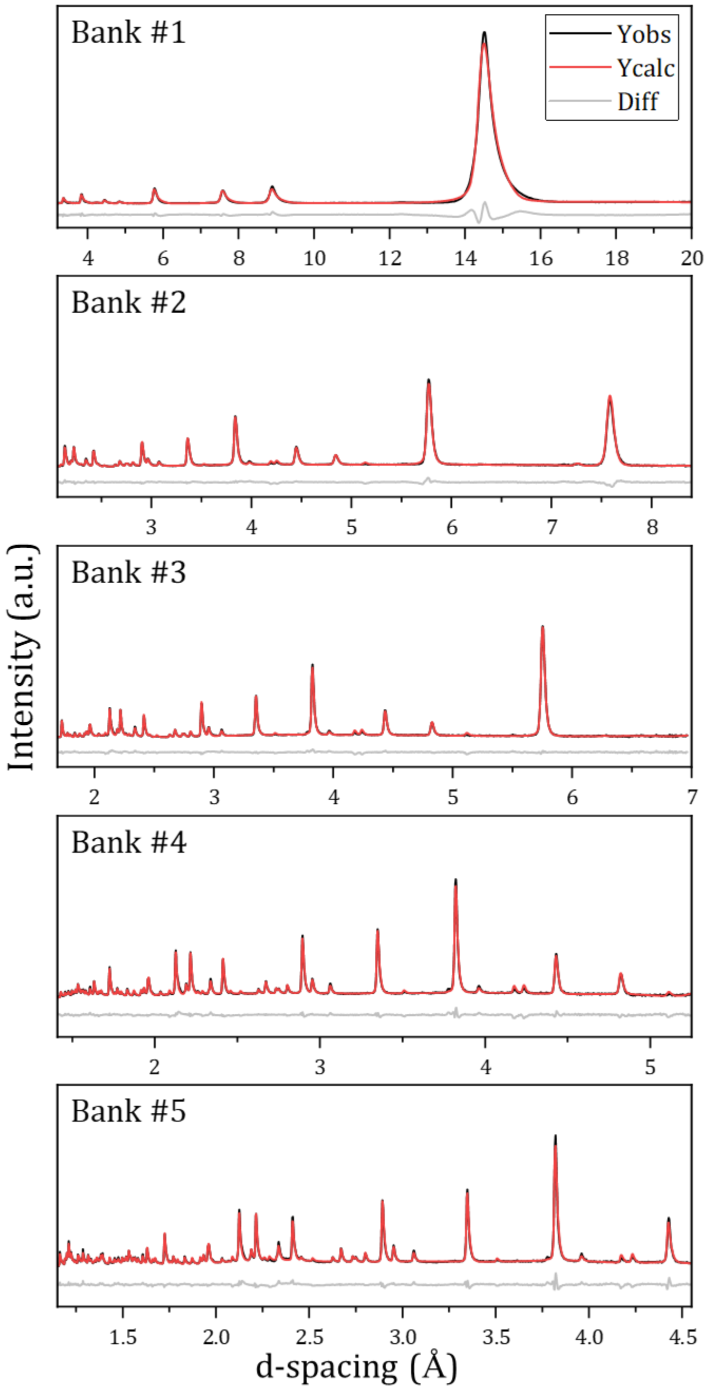

Figure S50. Neutron diffraction patterns and Rietveld refinement for activated Na-X (banks 1 to 5 from top to bottom).

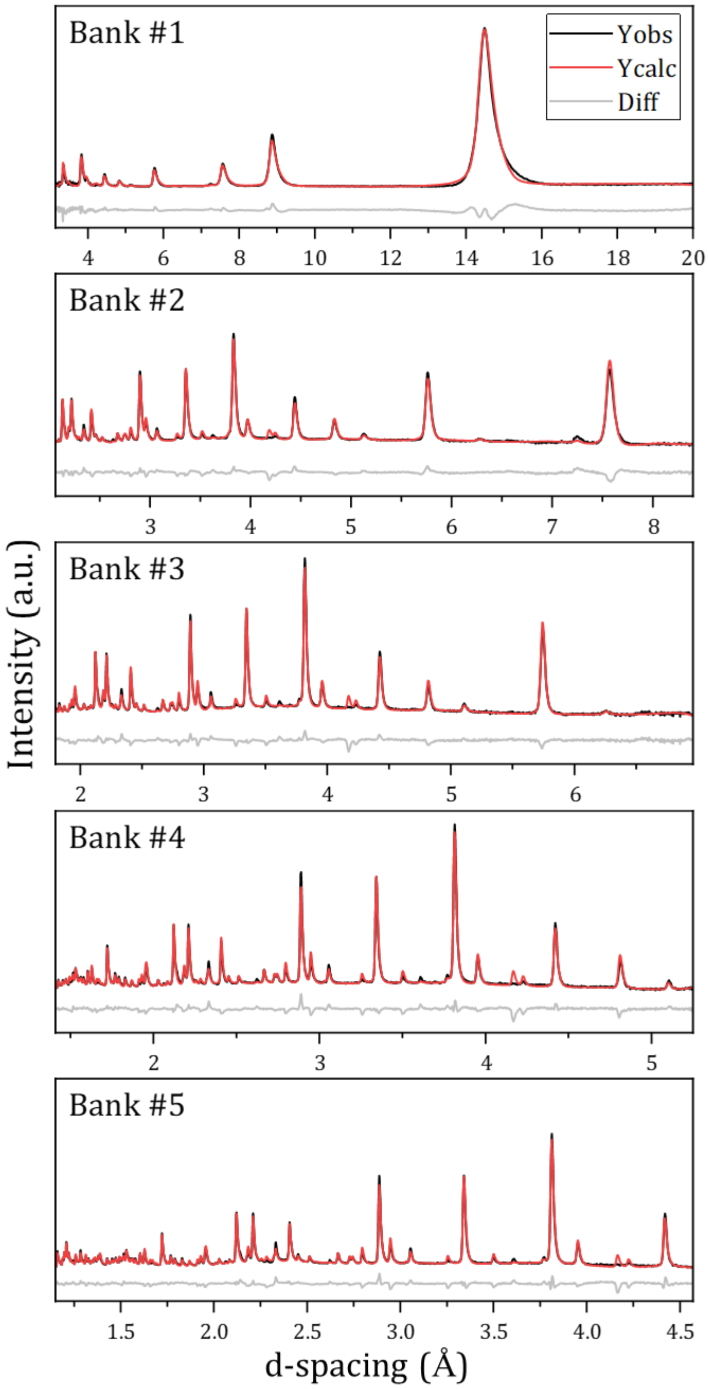

Figure S51. Neutron diffraction patterns and Rietveld refinement for CO_2-loaded Na-X (banks 1 to 5 from top to bottom).

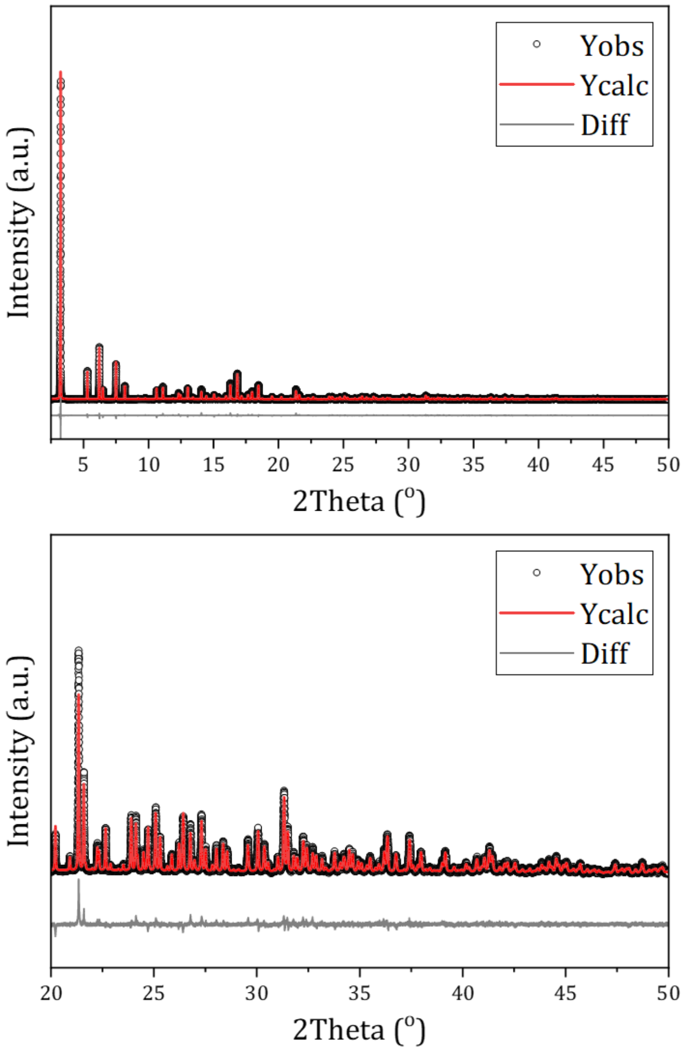

Figure S52. Synchrotron X-ray diffraction patterns and Rietveld refinement for activated Ba-X (up: 2.5–50°, down: 20–50°).

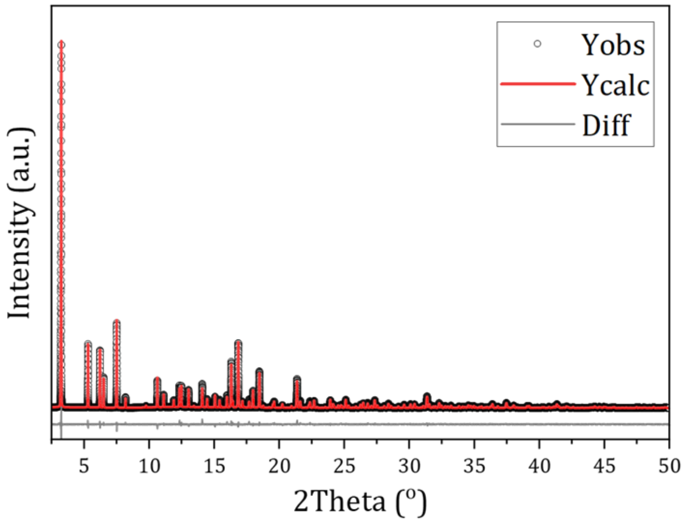

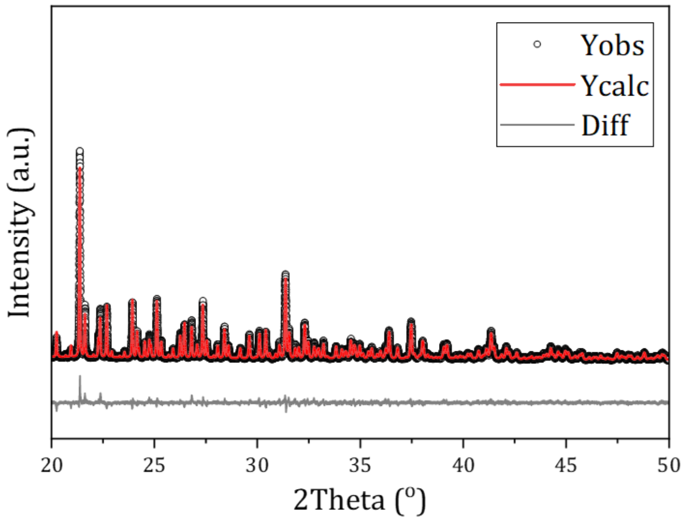

Figure S53. Synchrotron X-ray diffraction patterns and Rietveld refinement for CO_2-loaded Ba-X (up: 2.5–50°, down: 20–50°).

Tables

Table S1. Textual properties of M-X zeolites.

Zeolite	Li-X	Na-X	K-X	Cs-X	Mg-X	Ca-X	Ba-X
Exchange degree	100	100	100	80	86	99	98
BET surface area (m^2/g)	777	756	706	152	615	723	655
Total pore volume (mL/g)	0.352	0.349	0.346	0.115	0.374	0.401	0.323
Micropore area (m^2/g)	713	669	621	82	392	569	556
Micropore volume (mL/g)	0.260	0.244	0.228	0.039	0.140	0.217	0.203

Table S2. CO_2 adsorption data on M-X zeolites.

zeolites	Static CO_2 uptake at 298 K (mmol/g)					
	0.5 mbar	1 mbar	3 mbar	5 mbar	10 mbar	1 bar
Li-X	0.16	0.28	0.57	0.75	1.02	5.34
Na-X	0.44	0.69	1.17	1.42	1.80	5.44
K-X	0.29	0.40	0.71	0.92	1.28	5.35
Cs-X	0.07	0.09	0.15	0.18	0.24	0.80
Mg-X	0.05	0.07	0.15	0.21	0.32	3.65
Ca-X	0.47	0.59	0.83	0.97	1.19	4.71
Ba-X	0.43	0.71	1.32	1.65	2.09	4.46

Table S3. CO_2 adsorption data of M-X zeolites on per standard volume.

Zeolites	CO_2 absorbed on per cm^3		
	Static conditions (1 bar)	Static conditions (0.01 bar)	Dynamic conditions (10,000 ppm)
Li-X	184.4	35.2	18.5
Na-X	221.6	71.4	46.0
K-X	237.0	56.4	39.1
Cs-X	79.3	22.9	21.9
Mg-X	192.0	16.5	14.4
Ca-X	204.8	51.3	40.1
Ba-X	233.7	108.8	93.4

Table S4. Comparison of adsorbents for DAC of CO_2 under ambient conditions.

Adsorbent	Static uptake (mmol/g)	Dynamic uptake (mmol/g)	Ref
SIFSIX-3-Ni	2.0 (10 mbar)	1.7 (1%, dry)	1
NbOFFIVE-1-Ni	2.2 (10 mbar)<br>1.3 (0.4 mbar)	1.8 (1%, dry)<br>1.2 (1%, wet)	2
$Mg_2(dobdc)(N_2H_4)_{1.8}$	3.9 (0.4 mbar)<br>4.8 (10mbar)	4.2 (1000 ppm, dry)	3
SIFSIX-18-Ni-β	2.0 (10 mbar)<br>0.8 (1 mabr)	1.3 (3000 ppm, dry)<br>0.8 (3000 ppm, wet)<br>0.6 (1000 ppm, dry)<br>0.3 (1000 ppm, wet)	4
NbOFFIVE-1-Ni@PA	2.3 (10 mbar)<br>1.4 (0.4 mbar)	0.9 (400 ppm, wet)	5
$Zn\text{-}(ZnOH)_4(bibta)_3$	2.2 (0.4 mbar)	1.3 (395 ppm, dry)	6
SIFSIX-3-Cu	2.3 (10 mbar)	0.4 (1000 ppm, dry)	7
ZU-36-Ni	1.1 (0.4mbar)<br>1.6 (1 mbar)<br>2.4 (10 mbar)	0.5 (1%, dry)	8
Zn-CHA7-1.9W	/	0.2 (400 ppm, wet)	9
TRI-PE-MCM-41	1.0 (0.4 mbar)<br>1.6 (10 mbar)	0.9 (400 ppm, dry)	10
MIL-101 (Cr)-PEI-800	/	1.3 (400 ppm, dry)	11
Co-MOF (1a′)	1.4 (0.4 mbar)<br>5.7 (10 mbar)	1.1 (400 ppm, dry)	12
ZU-16-Co	1.1 (0.4 mbar)<br>2.6 (10 mbar)	0.37 (1%, dry)	
TIFSIX-3-Ni	0.7 (0.4 mbar)<br>1.8 (10 mbar)	/	13
dptz-CuTiF6	2.1 (10 mbar)	/	

NPEI-SIPs	1.7 (0.4 mbar)	1.1 (400 ppm, dry)<br>1.7 (400 ppm, wet)	
SBA-15	/	1.6 (400 ppm, dry)	14
PE-MCM-41	/	2.2 (400 ppm, dry)<br>2.9 (400 ppm, wet)	
Ba-X	0.4 (0.4 mbar)<br>0.7 (1 mbar)<br>2.1 (10 mbar)	1.8 (1%, dry)<br>1.6 (1%, wet)<br>1.2 (3000 ppm, dry)<br>1.0 (3000 ppm, wet)<br>0.7 (1000 ppm, dry)<br>0.4 (1000 ppm, wet)<br>0.3 (400 ppm, dry)	This work

Table S5. CO_2 adsorption data of M-X zeolites on per microporous surface area.

Zeolites	CO_2 absorbed on per 1000 m^2 microporous surface area		
	Static conditions (1 bar)	Static conditions (0.01 bar)	Dynamic conditions (10,000 ppm)
Li-X	7.48	1.43	0.75
Na-X	8.29	2.67	1.72
K-X	8.61	2.05	1.42
Cs-X	9.76	2.82	2.70
Mg-X	9.31	0.80	0.70
Ca-X	8.27	2.07	1.62
Ba-X	8.03	3.74	3.21

Table S6. Henry's law constants for CO_2 adsorption on M-X zeolites

Zeolites	K_H (mol kg^{-1} kPa^{-1})		Statistics		Q_{st}
	273 K	303 K	273 K	303 K	(kJ mol^{-1})
Li-X	19.89	4.64	0.985	0.990	33.7
Na-X	45.03	5.17	0.986	0.965	50.0
K-X	50.02	5.79	0.995	0.997	52.3
Mg-X	3.00	0.95	0.998	0.999	26.6
Ca-X	58.46	7.47	0.983	0.964	47.5
Ba-X	125.69	11.62	0.999	0.999	55.0

Table S7. Textual properties of M-Y zeolites.

Zeolites	Li-Y	Na-Y	K-Y	Cs-Y	Mg-Y	Ca-Y	Ba-Y
Exchange degree (%)	85	100	100	81	78	88	99
BET surface area (m^2/g)	741	710	661	426	788	671	685
Total pore volume (mL/g)	0.401	0.334	0.332	0.241	0.332	0.287	0.345
Micropore area (m^2/g)	668	652	599	352	738	631	593
Micropore volume (mL/g)	0.247	0.232	0.225	0.134	0.269	0.233	0.228

Table S8. Breakthrough data of M-Y zeolites in CO_2 capture

Adsorbent	Dynamic uptake (mmol/g)				Humidity
	10000 ppm	5000 ppm	3000 ppm	1000 ppm	
Li-Y	0.16	0.10	0.07	0.02	dry
Na-Y	0.24	0.18	0.09	0.03	dry
K-Y	0.40	0.25	0.16	0.07	dry
Cs-Y	0.83	0.71	0.51	0.18	dry
Mg-Y	0.23	0.16	0.08	0.03	dry
Ca-Y	0.26	0.19	0.16	0.10	dry
Ba-Y	0.99	0.79	0.61	0.43	dry

Table S9. Characteristics of oxygen charge on M-Y zeolites.

Zeolites	Unit cell composition	Intermediate electronegativity (S_{int})	Mean charge on oxygen atom (δ_O)
Li-Y	$Li_{53}(Al_{53}Si_{139}O_{384})$	3.597	0.340
Na-Y	$Na_{53}(Al_{53}Si_{139}O_{384})$	3.580	0.343
K-Y	$K_{53}(Al_{53}Si_{139}O_{384})$	3.430	0.375
Cs-Y	$Cs_{53}(Al_{53}Si_{139}O_{384})$	3.314	0.399
Mg-Y	$Mg_{26.5}(Al_{53}Si_{106}O_{384})$	3.985	0.258
Ca-Y	$Ca_{26.5}(Al_{53}Si_{106}O_{384})$	3.941	0.267
Ba-Y	$Ba_{26.5}(Al_{53}Si_{106}O_{384})$	3.865	0.283

Table S10. Crystal Data and Details of the Structure Determination for M-X.

Materials	Na-X	CO_2@Na-X	Ba-X	CO_2@Ba-X
Formula	$Na_{86}(Al_{86}Si_{106}O_{384})$	$Na_{86}(Al_{86}Si_{106}O_{384})(CO_2)_{37}$	$Ba_{43}(Al_{86}Si_{106}O_{384})$	$Ba_{43}(Al_{86}Si_{106}O_{384})(CO_2)_{68}$
Crystal System	Cubic	Cubic	Cubic	Cubic
Space group	*Fd-3m*	*Fd-3m*	*Fd-3m*	*Fd-3m*
a [Å]	24.994(3)	24.941(4)	25.24196(15)	25.20725(8)
V [Å 3]	15613(6)	15515(7)	16083.1(3)	16016.82(14)
Radiation type	Neutron Source, time-of-flight		Synchrotron X-ray λ = 0.826833 Å	
R_{wp}(%)	2.1728	2.0483	7.5304	6.7841
R_p(%)	1.6450	1.5601	5.6683	5.2529
R_{exp}(%)	0.4484	0.4432	3.1688	3.2669
GOF	4.8456	4.6209	2.3763	2.0765
CCDC	2164938	2164939	2164936	2164937

Reference

[1] Chen, K.-J.; Yang, Q.-Y.; Sen, S.; Madden, D. G.; Kumar, A.; Pham, T.; Forrest, K. A.; Hosono, N.; Space, B.; Kitagawa, S.; Zaworotko, M. J. Efficient CO_2 Removal for Ultra-Pure CO Production by Two Hybrid Ultramicroporous Materials. *Angew. Chem. Int. Ed.* **2018**, *57* (13), 3332–3336.

[2] Bhatt, P. M.; Belmabkhout, Y.; Cadiau, A.; Adil, K.; Shekhah, O.; Shkurenko, A.; Barbour, L. J.; Eddaoudi, M. A Fine-Tuned Fluorinated MOF Addresses the Needs for Trace CO_2 Removal and Air Capture Using Physisorption. *J. Am. Chem. Soc.* **2016**, *138* (29), 9301–9307.

[3] Liao, P. Q.; Chen, X. W.; Liu, S. Y.; Li, X. Y.; Xu, Y. T.; Tang, M.; Rui, Z.; Ji, H.; Zhang, J. P.; Chen, X. M. Putting an Ultrahigh Concentration of Amine Groups into a Metal-Organic Framework for CO_2 Capture at Low Pressures. *Chem. Sci.* **2016**, *7* (10), 6528–6533.

[4] Mukherjee, S.; Sikdar, N.; O'Nolan, D.; Franz, D. M.; Gascón, V.; Kumar, A.; Kumar, N.; Scott, H. S.; Madden, D. G.; Kruger, P. E.; Space, B.; Zaworotko, M. J. Trace CO_2 Capture by an Ultramicroporous Physisorbent with Low Water Affinity. *Sci. Adv.* **2019**, *5* (11), eaax9171.

[5] Guo, M.; Wu, H.; Lv, L.; Meng, H.; Yun, J.; Jin, J.; Mi, J. A Highly Efficient and Stable Composite of Polyacrylate and Metal-Organic Framework Prepared by Interface Engineering for Direct Air Capture. *ACS Appl. Mater. Interfaces* **2021**, *13* (18), 21775–21785.

[6] Bien, C. E.; Chen, K. K.; Chien, S. C.; Reiner, B. R.; Lin, L. C.; Wade, C. R.; Ho, W. S. W. Bioinspired Metal-Organic Framework for Trace CO_2 Capture. *J. Am. Chem. Soc.* **2018**, *140* (40), 12662–12666.

[7] Shekhah, O.; Belmabkhout, Y.; Chen, Z.; Guillerm, V.; Cairns, A.; Adil, K.; Eddaoudi, M. Made-to-Order Metal-Organic Frameworks for Trace Carbon Dioxide Removal and Air Capture. *Nat. Commun.* **2014**, *5*, 4228.

[8] Zhang, Z.; Ding, Q.; Peh, S. B.; Zhao, D.; Cui, J.; Cui, X.; Xing, H. Mechano-Assisted Synthesis of an Ultramicroporous Metal-Organic Framework for Trace CO_2 capture. *Chem. Commun.* **2020**, *56* (56), 7726–7729.

[9] Fu, D.; Park, Y.; Davis, M. E. Zinc Containing Small-Pore Zeolites for Capture of Low Concentration Carbon Dioxide. *Angew. Chem. Int. Ed.* 2021, 60, 2–9.

[10] Belmabkhout, Y.; Serna-Guerrero, R.; Sayari, A. Adsorption of CO_2-Containing Gas Mixtures over Amine-Bearing Pore-Expanded MCM-41 Silica: Application for Gas Purification. *Ind. Eng. Chem. Res.* 2010, 49, 359–365.

[11] Darunte, L.A.; Oetomo, A. D.; Walton, K. S.; Sholl, D. S.; Jones, C. W. Direct Air Capture of CO2 Using Amine Functionalized MIL-101(Cr). *ACS Sustainable Chem. Eng.* 2016, 4, 5761–5768.

[12] Hu, P.; Liu, H.; Wang, H.; Zhou, J.; Wang, Y.; Ji, H. Synergic Morphology Engineering and Pore Functionality within a Metal–Organic Framework for Trace CO_2 Capture. *J. Mater. Chem. A*, 2022, 10, 881–890.

[13] Zhang, Z.; Ding, Q.; Cui, J.; Cui, X.; Xing H. High and Selective Capture of Low-Concentration CO_2 with an Anion-Functionalized Ultramicroporous Metal-Organic Framework. *Sci. China Mater.* 2021, 64, 691–697.

[14] Rim, G.; Feric, T. G.; Moore, T.; Park A. -H. A. Solvent Impregnated Polymers Loaded with Liquid-Like Nanoparticle Organic Hybrid Materials for Enhanced Kinetics of Direct Air Capture and Point Source CO_2 Capture. *Adv. Funct. Mater.* 2021, 31, 2010047.
